# Pectin Methylesterases: Cell Wall Remodeling Proteins Are Required for Plant Response to Heat Stress

**DOI:** 10.3389/fpls.2018.01612

**Published:** 2018-11-06

**Authors:** Hui-Chen Wu, Victor P. Bulgakov, Tsung-Luo Jinn

**Affiliations:** ^1^Department of Biological Sciences and Technology, National University of Tainan, Tainan, Taiwan; ^2^Institute of Biology and Soil Science, Far Eastern Branch of the Russian Academy of Sciences, Vladivostok, Russia; ^3^Department of Life Science, Institute of Plant Biology, National Taiwan University, Taipei, Taiwan

**Keywords:** cell wall remodeling, heat stress response, guard cell wall, pectin, pectin methylesterase

## Abstract

Heat stress (HS) is expected to be of increasing worldwide concern in the near future, especially with regard to crop yield and quality as a consequence of rising or varying temperatures as a result of global climate change. HS response (HSR) is a highly conserved mechanism among different organisms but shows remarkable complexity and unique features in plants. The transcriptional regulation of HSR is controlled by HS transcription factors (HSFs) which allow the activation of HS-responsive genes, among which HS proteins (HSPs) are best characterized. Cell wall remodeling constitutes an important component of plant responses to HS to maintain overall function and growth; however, little is known about the connection between cell wall remodeling and HSR. Pectin controls cell wall porosity and has been shown to exhibit structural variation during plant growth and in response to HS. Pectin methylesterases (PMEs) are present in multigene families and encode isoforms with different action patterns by removal of methyl esters to influencing the properties of cell wall. We aimed to elucidate how plant cell walls respond to certain environmental cues through cell wall-modifying proteins in connection with modifications in cell wall machinery. An overview of recent findings shed light on PMEs contribute to a change in cell-wall composition/structure. The fine-scale modulation of apoplastic calcium ions (Ca^2+^) content could be mediated by PMEs in response to abiotic stress for both the assembly and disassembly of the pectic network. In particular, this modulation is prevalent in guard cell walls for regulating cell wall plasticity as well as stromal aperture size, which comprise critical determinants of plant adaptation to HS. These insights provide a foundation for further research to reveal details of the cell wall machinery and stress-responsive factors to provide targets and strategies to facilitate plant adaptation.

## Introduction

Plants face challenges of extreme environmental conditions, which include various abiotic and biotic stresses, all of which exert adverse effects on plant growth and development. Being sessile organisms, plants cannot move to favorable environments; accordingly, they have developed a remarkable number of strategies to mitigate environmental impacts. Global climate change constitutes one of the most detrimental stresses to plants because it is driving an increase in ambient temperatures, which, according to the Intergovernmental Panel on Climate Change prediction (IPCC, 2012), are expected to be 2–5°C higher than the current temperatures by the late twenty-first century. Extremely high temperatures can cause devastating damage to crops and reduce crop production; however, plant cells have been shown to have elaborate systems to respond to a variety of challenges, including HSR, which can improve crop yield under climate change conditions.

Acquired thermotolerance in plants consists of translating an initial moderate temperature increment into molecular defenses against subsequent extreme temperatures, such as preventing and repairing damage to heat-labile proteins and membranes (Larkindale and Vierling, 2008). HSR is characterized by the induction of a large set of HSPs, many of which comprise chaperone proteins that assist in protein folding and protect cellular homeostasis against heat and other stress stimuli (Morimoto, 2008). In addition to the induction of HS-responsive genes, the modification of biophysical properties of the cell wall may represent a key component in responding to environmental injuries. For example, HS at 37°C can generate changes in cell wall polymers in coffee (*Coffea arabica*) leaves, resulting in ∼50% decrease in pectin and 40% increase in hemicellulose (Lima et al., 2013). Thus, the modulation of plant cell walls, which comprise a dynamic and interconnected network consisting of a heterogeneous matrix with diverse biochemical and mechanical properties, has emerged as an important strategy in plant stress responses.

Pectic polysaccharides are highly heterogeneous polymers involved in the control of cell wall porosity and constitute the major adhesive material between cells (Willats et al., 2001). When the cell is challenged with stress conditions, specific transcriptional responses affect the production of certain cell wall proteins, leading to crucial changes in cell wall architecture (Klis et al., 2006). Pectin modification is catalyzed by a large enzyme family of PMEs that reside in the cell wall and which modulate apoplastic Ca^2+^ content in response to stresses for both the assembly and disassembly of the pectic network (Micheli, 2001; Wu and Jinn, 2010; Wu et al., 2010). Thus, the maintenance of cell wall integrity is tightly controlled and strictly coordinated with the stress response in plant cells. Pectins have been identified as key elements in plant responses to either heat or cold temperature stress in various species such as winter oilseed rape (*Brassica napus* var. *oleifera*), bromeliad (*Nidularium minutum*), Arabidopsis (*Arabidopsis thaliana*), rice (*Oryza sativa*), soybean (*Glycine max*), and coffee (Wu and Jinn, 2010; Carvalho et al., 2013; Huang et al., 2017). However, the cell wall factors that contribute to the development of plant thermotolerance remain largely unknown.

A previous review revealed that PME-mediated changes in the cell wall have played a role in various vegetative and reproductive developmental processes in Arabidopsis and other dicotyledons (Wolf et al., 2009). The DM of HGAs can be controlled by PMEs that have the capacity to remove methyl ester groups to contribute to the intercellular adhesion during plant development and stress responses (Wu et al., 2010; Le Gall et al., 2015). Thus, the DM constitutes a key element in the control of the wall stiffness and hydration status of the pectic matrix during abiotic stresses. PMEs exhibit a potential for the development of thermotolerance by maintaining apoplastic Ca^2+^ homeostasis (Wu et al., 2010). Heat-activated PME activity is involved in the cell-wall localization of Ca^2+^, i.e., with the removal of apoplastic Ca^2+^ that participates in HS signaling to induce HSP expression and cell-wall remodeling to retain plasma membrane integrity, thus preventing cellular content leakage and conferring thermoprotection (Wu and Jinn, 2010). Furthermore, through the identification of Arabidopsis *PME34* mutant plants, it was verified that the thermotolerance impairment of *pme34* was independent from the expression of HS-responsive genes; whereas PME34 functions in controlling stomatal movements and in regulating the flexibility of the guard cell wall required for heat response (Huang et al., 2017).

Little is known about the dynamics of the pectin matrix in the regulation of the impact of stress on plants. In previous research, we focused on the role of the PME, which is intrinsically involved in the modification of cell wall components in response to HS; and most recently demonstrated that the dynamic network of cell wall remodeling proteins with enzymatic activity is crucially important for cell wall tolerance to HS (Wu and Jinn, 2010; Wu et al., 2010, 2017; Huang et al., 2017). The present review, therefore, describes the most recent findings regarding cell wall remodeling and HSR as well as specific issues with the characterization of PME. In addition, this review also highlights the diversity of their roles during plant development and in response to diverse abiotic stresses, particularly to HS.

## Regulation of the Heat Stress Response in Plants

Heat stress causes a broad spectrum of cellular damage through the extensive denaturation and aggregation of proteins, and by modifying membrane permeability and fluidity, which subsequently disrupts the balance of metabolic processes. In nature, such HS conditions may be chronic or recurring, or both (Bäurle, 2016); therefore, plants have developed diverse systems to cope with recurring stress. HSR is a highly conserved stress response mechanism that reflects how plants respond and adapt to HS through improved thermotolerance. It defines all high temperature-related defense activities used in the cell to prevent damage and aggregation at the proteome level (Lindquist and Craig, 1988; Vierling, 1991).

**FIGURE 1:**
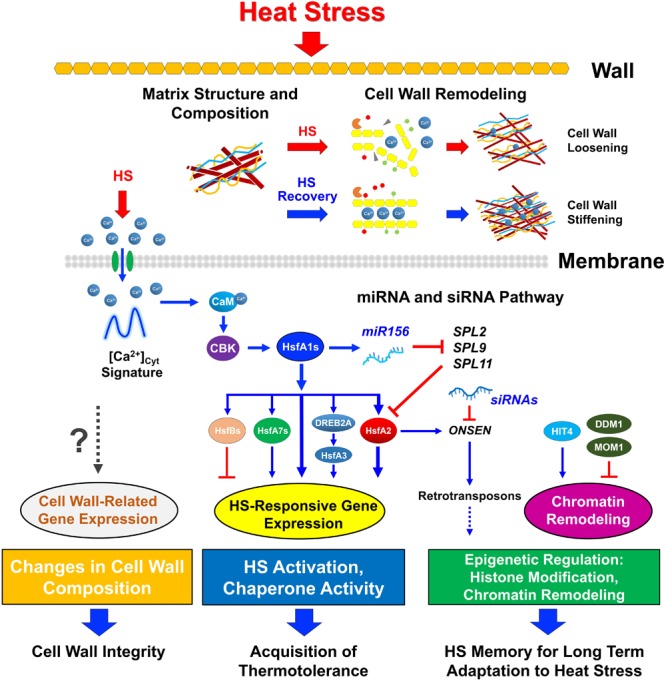
Integration of cell wall remodeling and the heat response network. Plant perception of heat involves several pathways in different compartments. The cell wall is the first protective barrier in plants that is exposed to heat. Heat stress (HS)-triggered pectin methylesterases (PME) activity, accompanied by Ca^2+^ mobilization from apoplastic sources, is involved in cell wall remodeling and is crucial for plant thermotolerance. During recovery time after HS, PME performs linear demethylesterification on highly esterified pectin residues and interacts with Ca^2+^ to form a pectate gel lawn, which causes cell-wall stiffening. During non-lethal HS, acidic PME acts randomly on pectin and promotes the action of endo-polygalacturonases (PG) to contribute to cell-wall loosening and the release of Ca^2+^ through Ca^2+^-permeable channels (green oval) in the plasma membrane, thus causing a transient increase in [Ca^2+^]_cyt_ oscillation. This is followed by induction of a Ca^2+^/calmodulin (CaM)-dependent pathway to activate the master HS regulator HsfA1s, which directly triggers HS-responsive transcription factors, including HsfA2, HsfA7s, HsfBs, and dehydration-responsive element-binding protein 2A for downstream *HsfA3* and *HSP* gene expression involved in the acquisition of thermotolerance. Histone modification and several epigenetic regulators, including small RNAs and transposons, are involved in the HSR and HS memory. MicroRNA156 targets the *SQUAMOSA* promoter-binding protein-like gene family, which downregulates HS-inducible genes and therefore maintains the expression of *HsfA2* and *HSP* genes during recovery from HS for long-term adaptation to HS. The retrotransposon ONSEN, as a target of *HsfA1s* and *HsfA2*, can be modulated by siRNAs for the regulation of HS memory. Through heat-intolerant 4 (HIT4), HS can relax the silencing of transposons, whereas they can be silenced by deficient in DNA methylation 1 (DDM1) and Morpheus’ molecule 1 (MOM1). However, the HS-induced cell wall-related transcript profile needs to be further explored with regard to the maintenance and modification of cell wall integrity.

The induction of HSPs constitutes one of the best-characterized responses in the adaptation to elevated temperature and plays an important role in the acquisition of thermotolerance. Recently, epigenetic mechanisms have been found to play important roles in the regulation of HSR, including DNA methylation (Boyko et al., 2010; Folsom et al., 2014; Lämke and Bäurle, 2017), histone modification (Min et al., 2014), histone variants (Kumar and Wigge, 2010), ATP-dependent chromatin remodeling (Mlynárová et al., 2007), and siRNAs and miRNAs (Ito et al., 2011; Ballén-Taborda et al., 2013). For example, miRNA 156, 160, and 172 modulation of *HSP* gene induction is required for Arabidopsis thermotolerance (Khraiwesh et al., 2012; Lin et al., 2018). Accumulation of the heat-induced retrotransposon ONSEN, which is recognized by HS transcription factors HsfA1 and HsfA2 through its HSE, is required for the regulation of HS memory (Ito et al., 2011; Cavrak et al., 2014; Ohama et al., 2017). Arabidopsis HIT4 is a chromocenter-localized protein that functions as a regulator of stress-triggered chromatin re-organization that is essential for plant heat tolerance (Wang et al., 2013). Therefore, it appears as if the epigenetic control of heat-responsive gene expression is frequently utilized to prevent heat-related damages (Liu et al., 2015; Lämke and Bäurle, 2017; Ohama et al., 2017). Furthermore, the emerging evidence indicates that cell wall remodeling plays a crucial role in the response to HS through the activation of cell wall-related genes and alteration of cell wall compositions (Wu and Jinn, 2010; Wu et al., 2010, 2017; Huang et al., 2017). Thus, the modification of cell wall structures to enhance their functions to perceive and respond to multiple environmental stresses is crucial for plants by imparting stress endurance. We summarize the current knowledge regarding plant HSR with different aspects to integrate cellular compartments and signaling networks as addressed in Figure 1.

### Plant Heat Stress Transcription Factor-Mediated Heat Stress Transcriptional Network

**FIGURE 2:**
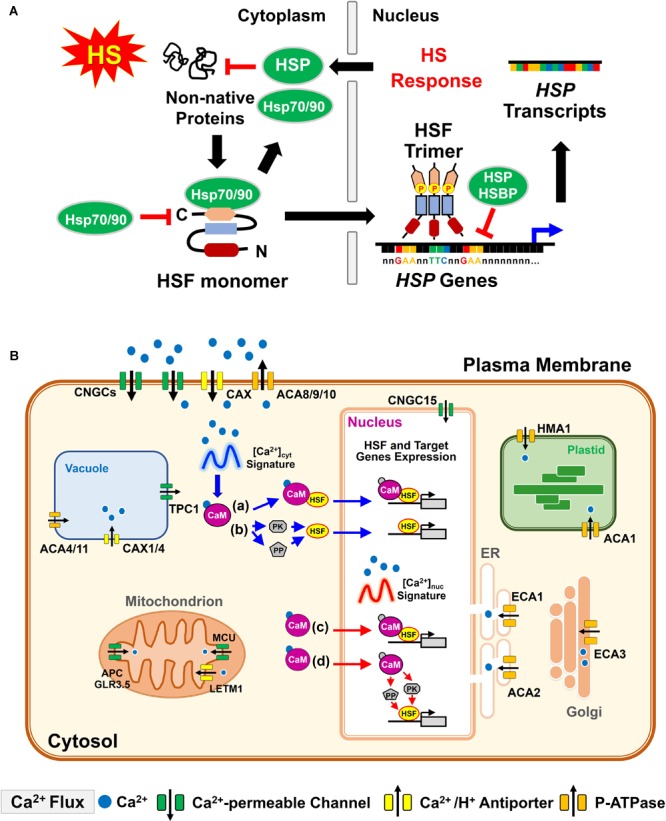
Basic function of plant HSF and HS-induced [Ca^2+^]_cyt/nuc_ oscillation interpretation by CaM in response to heat. **(A)** Under unstressed conditions, Hsp70/HspP90 can directly regulate the function of HSF by blocking its transcriptional activity. Upon HS, non-native proteins induce the conversion of monomeric HSF into an active trimeric form, which is phosphorylated and translocated into the nucleus. HSF trimer, with high-affinity DNA binding capacity to the HSE (5′-nGAAnnTTCnnGAAn-3′) of the *HSP* gene promoter region, activates *HSP* gene expression, whereas it is downregulated by the interaction of HSP and HSBP with the HSF trimer to attenuate HSR in plants. HSP production and relocation to the cytoplasm inhibits non-native protein misfolding and aggregation. **(B)** Cellular Ca^2+^ transport is tightly controlled within all membrane-bound organisms during heat stress. An increase in [Ca^2+^]_cyt_ is manifested by Ca^2+^ influx to the cytosol, mediated by Ca^2+^-permeable ion channels, either from the apoplast across the plasma membrane, or from intracellular stores such as the endoplasmic reticulum or vacuole. In contrast, Ca^2+^-ATPases and the Ca^2+^/H^+^ antiporter systems are responsible for Ca^2+^ extrusion out of the cytosol. HS-elevated Ca^2+^ occurs from apoplast entry to the cytosol or nucleus (either diffused from the cytosol or released from nuclear Ca^2+^ reservoirs). The CaM responds to the elevation of [Ca^2+^]_cyt_ signature to modulate the activity of numerous target proteins. (a) and (c) The Ca^2+^/CaM complex interacts with the HS transcription factors (HSFs) and modulates either HSF DNA-binding or transcriptional activities. (b,d) The Ca^2+^/CaM complex regulates the activation of HSF by modulating the phosphorylation status. The regulation is achieved by CaM-binding protein kinase (PK) or CaM binding protein phosphatase (PP). (c,d) CaM recognizes a high frequency and magnitude of the cytosolic Ca^2+^ signature and is translocated into the nucleus for responding to the nuclear [Ca^2+^] ([Ca^2+^]_nuc_) to bind or regulate the status of HSF phosphorylation in the nucleus. ACAs, autoinhibited Ca^2+^-ATPases; APC, adenine nucleotide/phosphate carrier; CAXs, Ca^2+^/H^+^ cation antiporters; CNGC, cyclic nucleotide-gated ion channels; ECAs, ER-type calcium ATPases; GLR3.5, glutamate receptor 3.5; HMA1, heavy metal translocating P-type ATPase; LETM1; leucine zipper-EF-hand-containing transmembrane protein 1; MCU, mitochondrial calcium uniporter; TPC1, two-pore voltage-gated channel 1.

The inherent ability of plants to tolerate temperatures above those that are optimal for growth is termed basal (or intrinsic) thermotolerance (Larkindale et al., 2005). Plants also have the ability to acquire tolerance to otherwise lethal HS, referred to as acquired thermotolerance (or HS priming). Specifically, mild HS primes a plant to subsequently withstand or to acclimate to high temperatures that would otherwise be lethal to an unadapted plant (Mittler et al., 2012). This priming response of plants can be maintained over several days after mild HS and a return to normal growth conditions; which is referred to as the maintenance of acquired thermotolerance or HS memory (Charng et al., 2006, 2007; Stief et al., 2014). However, the molecular mechanisms involved in plant HS-priming and HS-memory remain largely unknown, especially for HS memory (Bäurle, 2016). In brief, heat stress transcription factors (HSFs) act as central regulators of HS priming by recognizing the conserved HSE in the promoter of the genes encoding HSP (Scharf et al., 1990), which in turn guard the proteome from misfolding and aggregation under heat conditions. The HSBP is a negative regulator of HSR through the interaction with HSF and thus dissociates trimeric HSFs for the attenuation of HSR (Satyal et al., 1998; Fu et al., 2002; Hsu et al., 2010; Rana et al., 2012). The regulation of HSFs in response to HS is illustrated in Figure 2A.

Four homologs of HsfA1 (HsfA1a, b, d, e) play roles of master regulators for acquired thermotolerance in Arabidopsis (Liu et al., 2011). HsfA2 has been shown to act as a secondary regulator under the control of HsfA1s to trigger a transcriptional cascade for the induction of early and late HS-responsive genes (Busch et al., 2005; Charng et al., 2007). Arabidopsis HsfB1 can act as a transcriptional repressor during the attenuation of HSR, whereas tomato (*Solanum lycopersicum*) HsfB1 possesses both coactivator and repressor functions (Bharti et al., 2004; Ikeda et al., 2011). Two major regulators of HSR, HsfA1s and dehydration-responsive element-binding protein 2A, are controlled by other regulatory factors such as NF-YC10, Hsp90, Hsp70, and small HSP (sHSP) (Hahn et al., 2011; Sato et al., 2014), with regard to their activation or inactivation in the early HSR or an unstressed condition. In addition, phytohormone ABA is also involved in HS signaling through the regulation of HsfA6b for plant thermotolerance (Huang et al., 2016). In yeast (*Saccharomyces cerevisiae*), Hsf1 in collaboration with protein kinase PKC1 regulates heat-induced cell-wall genes, including *CWP1, SPI1, HOR7, YGP1*, and *ZEO1*, to mediate maintenance of cell-wall integrity under HS (Imazu and Sakurai, 2005). Thus, yeast Hsf1 plays a role not only in the induction of *HSPs* expression but also in the induction of a set of cell-wall genes involved in cell-wall formation and remodeling to prevent cell lysis at high temperature. These data raise the question whether plant HSFs function as yeast Hsf1 involved in cell wall remodeling. Further work is required to determine the role of HSFs encoding a variety of other proteins that specifically function in plant cell-wall organization under HS.

### Ca^2+^/Calmodulin-Mediated Heat Stress Signaling

Despite the ubiquitous nature of the HSR, little is known about how plants sense an elevating temperature to transmit a signal that results in HSP induction and acquired thermotolerance. One candidate molecule that serves as a second messenger during HS signaling is calcium (Ca^2+^), a ubiquitous signal in eukaryotic cells. Ca^2+^ signals were shown to manifest through transient changes of spatio-temporal patterns of free cytosolic Ca^2+^ content ([Ca^2+^]_cyt_) arising from the flux of Ca^2+^ into the cytosol, defining the so-called Ca^2+^ signature (Dodd et al., 2010). A stress-induced change in [Ca^2+^]_cyt_ might constitute one of the primary transduction mechanisms whereby gene expression and biochemical events are altered to adapt to environmental stresses (Monroy and Dhindsa, 1995). The rise time, peak value, and duration of the decay back to resting levels of Ca^2+^ transients generated by early events have been implicated in controlling different transduction processes, including changes in gene expression (Dolmetsch et al., 1997; Kim et al., 2009). Depending on the specific activation properties, Ca^2+^ channels, Ca^2+^-ATPases, and Ca^2+^/H^+^ antiporters as modulators of Ca^2+^ shape the parameters and spatial characteristics of the Ca^2+^ flux, resulting in distinct a Ca^2+^ signature in response to different stress stimuli (Demidchik and Maathuis, 2007) (Figure 2B).

The stress-induced intracellular Ca^2+^ levels can be transmitted and sensed by a toolkit of Ca^2+^-binding proteins such as CaMs and their related-proteins, such as CaM-like proteins, calcineurin B-like proteins, and Ca^2+^-dependent protein kinases, for downstream responses. CaMs are highly conserved, consisting of two globular domains, each with two Ca^2+^-binding EF-hand motifs, and are considered to be multifunctional proteins. These proteins mostly act as general transducers of Ca^2+^-mediated signal cascades in eukaryotes submitted to various developmental and external stimuli. It was previously suggested that the transduction of environmental signals through *CaM* gene expression occurs in part by the elevation of [Ca^2+^]_cyt_ levels (Braam and Davis, 1990). In orchard grass (*Dactylis glomerata*), DgHsp70, a homolog of cytosolic Hsp70, can bind to Arabidopsis CaM2 in the presence of Ca^2+^, whereas negative regulation of DgHsp70 decreases the ATPase and foldase activities via Ca^2+^/CaM binding (Cha et al., 2012). Furthermore, CaM is involved in HSR through the interaction with cytosolic maize (*Zea mays*) Hsp70 and sorghum (*Sorghum bicolor*) Hsp90 (Sun et al., 2000; Virdi et al., 2009). Increasing evidence indicates that CaM plays a crucial role in HS responses that lead to an elevation of [Ca^2+^]_cyt_ signaling in various species (Gong et al., 1998; Liu et al., 2003, 2005; Wu et al., 2012). In wheat, *CaM1-2* gene expression increases after HS at 37°C for 10 min and reaches its peak expression after 20 min HS exposure, as determined by northern analysis (Liu et al., 2003). In moss *Physcomitrella patens*, a [Ca^2+^]_cyt_ elevation for 20 min was induced by HS via putative plasma membrane Ca^2+^-permeable channels (Saidi et al., 2009).

The elevated [Ca^2+^]_cyt_ and CaM can directly modulate the DNA-binding activity of HSF to HSE, suggesting that they are involved in the expression of *HSP* genes through the regulation of HSF (Mosser et al., 1990; Li et al., 2004). Arabidopsis signal responsive 1–6 genes (*SR1* to *SR6*), a Ca^2+^/CaM-binding transcription factor, play roles in transcription activation through specific binding to a “CGCG box” (A/C/G)CGCG(G/T/C) in the promoter of genes that are involved in multiple signal transduction pathways, including HSR in plants (Yang and Poovaiah, 2002). CaM is involved in the modulation of transcription factors either through direct interaction with basic helix-loop-helix domains, or by the control of kinase-mediated phosphorylation (Corneliussen et al., 1994; Corcoran and Means, 2001). In transgenic Arabidopsis, reporter *GUS* gene expression that is directed by the *Hsp18.2* promoter was shown to be affected by CaCl_2_ and CaM antagonists (Liu et al., 2005). Arabidopsis CBK3, by phosphorylating HsfA1a, enhances the binding activity to HSE, which promotes activation of *HSF* and *HSP* gene expression. Protein phosphatases, such as Arabidopsis PP7 are regulated by CaM that is dependent upon Ca^2+^-CaM binding, with the *pp7* mutation resulting in a reduction in acquired thermotolerance (Liu et al., 2007). We identified the rice *OsCaM1-1*, whose expression resembles the biphasic [Ca^2+^]_cyt_ signal, and showed that overexpression of *OsCaM1-1* induced the expression of Arabidopsis Ca^2+^/HS-related *CBK3, PP7, HSF*, and *HSP* genes, and enhanced intrinsic thermotolerance in transgenic Arabidopsis (Wu et al., 2012). Thus, OsCaM1-1 interprets the Ca^2+^ signal by the cytosolic Ca^2+^ concentration and by spatio-temporal Ca^2+^ parameters under HS. Furthermore, OsCaM1-1 contains potential miRNA168a and miRNA408 target sites, and both miRNAs harbor HSE, which may regulate transcription of these miRNAs in response to HS (Wu and Jinn, 2012).

Extracellular CaM was found to be involved in the initiation of pollen germination and tube growth by a heterotrimeric G protein in the cellular signaling process in lily (*Lilium longiflorum*) pollen (Ma et al., 1999). However, the functions of apoplastic CaM are still poorly understood in plant cells. In *Cedrus deodara*, apoplastic CaM maintained the tip-focused Ca^2+^ gradient and modulated the distribution of pectins during pollen tube growth (Wang et al., 2013). Apoplastic CaM contributed to Ca^2+^ homeostasis and cell wall remodeling during pollen development. Thus, the interaction between Ca^2+^ and apoplastic CaM may play a central role in the maintenance of Ca^2+^ gradients for cell-wall modeling. Arabidopsis NPG1 is a pollen-specific CaM-binding protein that interacts with PLLs, suggesting NPG1 may modify the pollen cell-wall through the interaction with PLLs (Shin et al., 2014). In addition, the largest releasable pool of Ca^2+^ is localized in the cell wall, reaching approximately 60–75% of the total tissue Ca^2+^ content (Demarty et al., 1984). Thus, apoplastic Ca^2+^ is essential for the control of cell integrity, cell wall cohesion, and plasma membrane permeability (Hirschi, 2004). It has been suggested that the increased [Ca^2+^]_cyt_ elevation observed in transformed tobacco (*Nicotiana tabacum*) seedlings during HS arises from both apoplastic and cytosolic sources (Gong et al., 1998). Potato (*Solanum tuberosum*) plant growth under HS can persist at specific levels of Ca^2+^ in the root, providing insight into the mechanism by which the zone of root Ca^2+^ may modulate plant response to HS (Kleinhenz and Palta, 2002). In moss (*Physcomitrella patens*), a specific Ca^2+^-permeable channel in the plasma membrane, which regulated heat-inducible Ca^2+^ influx, thereby leading to HSR (Saidi et al., 2009). Moreover, the recovery of HS-released Ca^2+^ is essential for the acquisition of thermoprotection to mitigate lethal HS injury both in soybean and rice seedlings (Wu and Jinn, 2010; Wu et al., 2010).

Notably, the cleavage of apoplastic Ca^2+^ bridges between pectic carboxyl groups that were created by PMEs is considered to play an important role in cell wall remodeling because it retains cell integrity during HS by preventing the plasma membrane from tearing away from the cell wall (Wu and Jinn, 2010; Wu et al., 2010). Thus, acquired thermotolerance is reported to critically depend on a preceding Ca^2+^ transient through the plasma membrane so that the HSR is regulated by the transient entry of apoplastic Ca^2+^ (Saidi et al., 2009; Wu and Jinn, 2012; Wu et al., 2012). Plant cells can monitor the functional integrity of cell walls, with the maintenance of cell wall integrity being an important process to relieve cellular stresses.

## Cell Wall Remodeling in Heat Response

### Plant Cell Wall Basics

**FIGURE 3:**
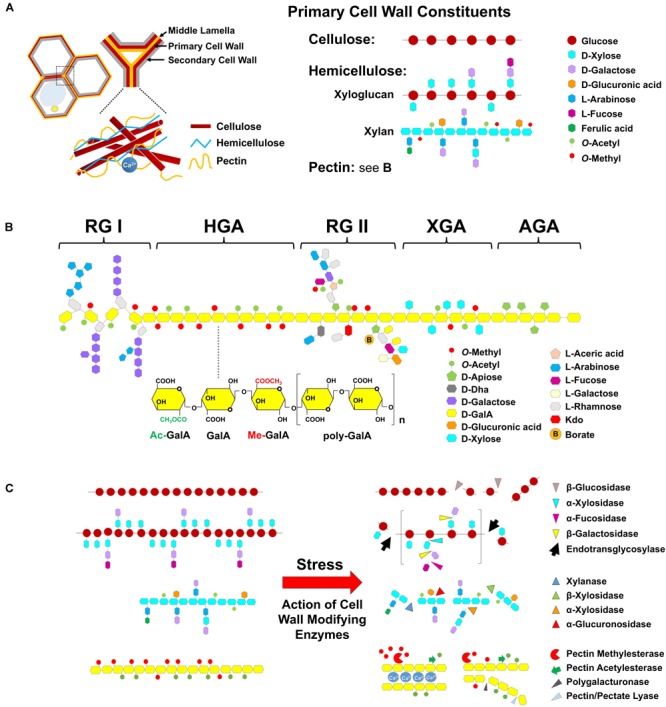
Cell wall composition and enzymatic modification in response to heat. **(A)** The cell wall is a complex structure that is composed of cellulose and non-cellulosic neutral polysaccharides embedded in a pectin matrix. Pectins are located in the middle lamella and primary and secondary cell wall. Major primary cell wall are constituted of cellulose microfibrils (multiple chains of β-glucose with β-1,4 glycosidic bonds) which are cross-linked to hemicelluloses and to pectin. Xyloglucan is a major hemicellulose molecule that is composed of β-1,4-linked glucose residues with α-1,6-linked xylosyl side chains. In turn, these side chains can be decorated with either galactose, or fucose residues to create a complex pattern of branches. Xylan consists a backbone of β-1,4-linked xylose (Xyl) residues that can be substituted with glucuronic acid and/or arabinose. Additional substitutions such as acetyl and methyl groups can be also presented. And **(B)** pectins are highly complex class of polysaccharides that comprise galacturonic acid-rich, consisting of five major classes, namely: homogalacturonan (HGA), rhamnogalacturonan I (RG-I), rhamnogalacturonan II (RG-II), xylogalacturonan (XGA), and apiogalacturonan (AGA) form a structurally diverse glue which provides stiffness or flexibility relying on the chemical modification. **(C)** Based on the action of hydrolysis and substrate specificity, the degradation of cellulose is cleaved by β-glucosidase into two molecules of glucose; for breaking down hemicellulose, xyloglucan endotransglycosylase/hydrolase (XTH), and expansin proteins (not shown) associated with disassembly of cellulose and xyloglucan matrix may play a role in the cell wall remodeling in different aspects of plant development and stress responses. Xylanase is responsible for degrading xylan by cleaving β-1,4 xylose linkages in the backbone. β-xylosidases cleave xylose from the non-reducing end of the xylan chain, and glucuronidases cleave the α-1,2 linked glucuronic acid, and α-arabinosidases cleave the α-1,2 and α-1,3 linked arabinose from the backbone. Pectinolytic enzymes such as PME, PAE, PG, PL, and Arabinanase by hydrolysis of pectic substances, are important for cell wall remodeling. The HGA, a polysaccharide of α-1,4-linked galacturonic acid (GalA) residues, is the predominant form of pectin. A critical feature of HGA that influences its properties is the methyl-esterification and acetylation of specific carbons on GalA during synthesis of the backbone. HGA is de-methylesterified by the activity of PME, which results in random and contiguous patterns of free carboxylic residues. De-methyl-esterification randomly releases protons, which become a target for pectin-degrading enzymes such as PG, which act by hydrolyzing the α-1,4 link between GalA. The contiguous de-methylesterified HGA binds with Ca^2+^ to induce gel formation, which can rigidify the cell wall.

The plant cell wall is a sophisticated structure formed by a complex mixture of cell wall polymers, such as polysaccharide-rich polymers, proteins, and pectin matrix that are assembled into a rigid, flexible, and dynamically organized network (Wu et al., 2017). Plant cell walls are multilayered and consist of three sections, including the middle lamella, primary cell wall, and secondary cell wall. The middle lamella is a pectin layer to cement the bond between two adjoining cells. The heterogeneous mixture of wall composition and thickness of the cell wall may deviate absolutely, depending on the environmental conditions. The primary wall surrounds growing cells or cells capable of cell growth; whereas the secondary wall is a highly specialized and thickened structure containing lignin, which undergoes irreversible changes in many fully developed cells. Cellulose is composed of repeating glucose residues connected through β-1,4-D-glucan (β-glucan) bonds that are crossed intricately together to form microfibrils as the scaffold of the cell wall and interconnected by hemicelluloses (xyloglucans and xylans are the most abundant) and galacturonic-acid-rich pectins (Figure 3A).

Pectin, a highly structurally complex polysaccharide, constitutes the major component of primary cell walls for both monocots and dicots, and is important for both cellular adhesion and cell wall plasticity (Mohnen, 2008). For example, pectin makes up 35% of the primary cell wall in dicots and non-grass monocots, 2–10% of grass primary walls, and up to 5% of wood tissues (Mohnen, 2008). The middle lamella, a pectinaceous interface, depends on the formation of intermolecular links between pectin molecules and is important for the adhesion of neighboring cells (Jarvis et al., 2003). Pectins also present in the junction zone between cells within secondary walls in the xylem and fiber cells of woody tissue (Mohnen, 2008). Fiber length of angiosperms is determined by intrusive tip growth, which requires dissolution of the middle lamella, wall loosening between adjacent cells to create space for tip growing (Goulao et al., 2011), and therefore, the modification of pectin may be occurring during secondary wall growth of trees. Generally, pectinaceous polysaccharides have been defined into five classes (Ridley et al., 2001; Caffall and Mohnen, 2009; Harholt et al., 2010), including HGA, RG-I and -II (RG-II), XGA, and AGA; presumably, these structural elements are linked covalently to form the pectin complex as shown in Figure 3B. It is generally believed that these pectic polysaccharides are covalently linked to, or tightly associated with other types of polysaccharides, since chemical treatments or digestion by pectin-degrading enzymes are required to isolate HGA, RG-I, and RG-II from each other and from cell walls (Nakamura et al., 2002; Coenen et al., 2007). The results support that a model of pectic polymers, HGA, RG-I, and RG-II are linked together during synthesis (Caffall and Mohnen, 2009). For instance, the HGA backbone can be hydrolyzed by PG to produce monomeric, dimeric, or oligomeric fragments; however, HGA, RG-I, and RG-II polysaccharides failed to resolve independently by size exclusion chromatography prior to fragmentation by PG digestion (York et al., 1996). Furthermore, the stretches of α-(1,4)-linked GalA of soybean soluble polysaccharides were found flanked by RG-I fragments, providing evidence that HGA and RG-I are directly connected through backbone residues (Nakamura et al., 2002). Similarly, it has been suggested that HGA is linked to xyloglucan through fragments of XGA that were not readily solubilized from walls unless treated with PG (Talmadge et al., 1973). Therefore, the backbone of HGA is covalently linked to RG-I and RG-II. It is also hypothesized to be crosslinked to xyloglucan or possibly other wall polymers *in muro*. In particular, HGA is a major component of pectin and has a conformational flexibility that can be influenced by growth, development, and environmental cues (Willats et al., 2001). HGA consists of a linear α-1,4-linked D-GalA homopolymer, which is the most abundant pectin-rich polysaccharide, constituting 65% of the total pectin. A critical feature of HGA that influences its properties is the methyl-esterification at C6-carboxyl and acetylation at C2 or C3 position by specific HGA-modifying enzymes, which belong to large multigenic families in all sequenced species (Gou et al., 2012; Sénéchal et al., 2014).

Owing to the characteristics of pectic matter which form hydrophilic colloids, it has been stated that the primary cell wall is plastic and soft. This component is crucial for cell growth and expansion, and is thought to contribute to cell wall structural integrity, cell adhesion, and signal transduction (Ochoa-Villarreal et al., 2012). In addition, the depolymerization of cellulose and hemicellulose, along with pectin, is particularly abundant and dynamic during plant development and stress responses in terms of modifying cell-wall polysaccharides. Consequently, enzymatic cleavage of the cross-linking polysaccharides by a set of cell wall-related enzymes including β-glucosidase, XET/XTH, and PME etc., which are believed to play a role in modulating cell wall plasticity, apparently mediate cell-wall integrity during plant development and stress responses (Figure 3C). The details are described below.

### Revealing the Mechanism of Cell Wall Integrity Maintenance in Response to Abiotic Stresses

It has been proposed that plants are able to respond to a spectrum of abiotic stress conditions due to modifications in cell-wall composition and structure to perform their respective functions for the maintenance of cell-wall integrity. However, our understanding of the mechanisms of stress-induced changes in wall composition and structure is still limited. Some cell wall-related genes have been shown to contribute directly to alter cell-wall composition to maintain cell-wall integrity under abiotic stress. Abiotic stress modified cell-wall constituents by CesA enzymes which alter cellulose biosynthesis (Wang et al., 2016), for instance, *AtCesA8/IRX1*, which encodes a subunit of a CesA complex to constitute part of the cell wall, plays an important role in drought and osmotic stress responses in Arabidopsis (Chen et al., 2005). Arabidopsis *SOS6* encodes a CesA-like protein (CSLD5) which has an important role in response to osmotic stress by regulating stress-induced ROS accumulation in plant cell walls (Zhu et al., 2010). In barley (*Hordeum vulgare*), a mutation in the *HvCslF6* gene that causes the loss of (1,3;1,4)-β-D-glucan reducing mixed-linkage glucan in primary cell wall yields mutants increasingly susceptible to chilling (Taketa et al., 2012). In leaves of tomato, β-glucosidase, which is responsible for degrading cellulose to free glucose molecules, is involved in the heat-stress response (Edreva et al., 2000). Additionally, β-glucosidase is likely involved in developing drought-tolerant wheat seedlings (cultivar Hong Mang Mai) by differentially changing cell-wall polysaccharides to favor drought tolerance (Konno et al., 2008).

In coffee, arabinose and galactose contents increased, whereas mannose, glucose, uronic acid, rhamnose, and fucose contents decreased after HS (Lima et al., 2013). The desiccated plant *Myrothamnus flabellifolius* had lower amounts of arabinoxylans than those in the hydrated plant, due to the increased association between cell-wall polymers under stress (Moore et al., 2006). Thus, the chemical profile and structural cell-wall polymers can be modified under HS. XET/XTH and EXP family members are involved in cell wall loosening and, therefore, in cell expansion for growth and development, as well as in the regulation of the plant responses under abiotic stress (Rose et al., 2002; Cosgrove, 2015). The overexpression of *Capsicum annuum XTH3* in tomato showed that increased salt tolerance involved cell-wall flexibility for alleviating stress effects (Choi et al., 2011). In maize, some cell wall-related genes were up-regulated under salinity stress, including *ZmXET1, ZmEXPA1, ZmEXPA3, ZmEXPA5, ZmEXPB1*, and *ZmEXPB2*, to hydrolyze and rejoin xyloglucan molecules during cell-wall extension (Li et al., 2014). When Arabidopsis plants were exposed to boron toxicity, the expression of genes that encode CesA (*CESA1, CESA4, CESA6*, and *CESA8*), and CesA-like *CSLB5, EXPs* (*EXPA5, EXP8*, and *EXPA14*) were reduced, while *PMEs* (*PME2* and *PME41*) showed a different expression pattern under boron stress and/or 24-epibrassinolide treatment (İşkil and Surgun-Acar, 2018). Heat-tolerant, thermal *Agrostis scabra, AsEXP1* was strongly induced by exposure to HS, is associated with thermotolerant grass germplasm (Xu et al., 2007). Overexpression of a Kentucky bluegrass (*Poa pratensis*) *PpEXP1* in tobacco exhibited a lesser extent of structural damage to cells resulted in enhanced HS tolerance. Thus, the *EXP* family may play more extensive and divergent effects on cell-wall integrity during stress responses. On the other hand, Arabidopsis *HOT2* encodes a CTL1 that is essential for tolerance to salt stress by preventing Na^+^ overaccumulation (Kwon et al., 2006). In Chinese cabbage (*Brassica rapa*), several genes encoding XTH proteins, β-glucosidase, CesA, EXP, extensin, glycosyl transferase, pectin esterase, and xylosidase, are up-regulated up to two–threefold following non-lethal temperature treatment at 37°C, which enables plants to survive a subsequent lethal temperature (Yang K. A. et al., 2006). Thus, these results provided evidence that cell wall-related proteins or enzymes are required for the cell-wall modifications involved in thermotolerance acquisition.

Recent studies have described that ROS and peroxidases are key players which initially cross-link phenolic compounds and extensins, causing cell-wall stiffening under drought stress (Tenhaken, 2014). In addition, OH^.^ radicals, which are able to cleave sugar bonds in polysaccharides, cause loosening of the cell wall similar to the action of EXPs or xyloglucan modifying enzymes (Renew et al., 2005). In the review by Houston et al. (2016), a broader consideration was made of multiple cell wall-related genes appearing to respond to a given stimulus, and a defined set of stress-responsive transcription factors involved in transcriptional regulation. However, a specific target for cell-wall modifications due to different stress responses has to be explored in detail, especially in distinct species.

### Enzymatic Modification of Cell Wall Structure and Integrity

It has been reported that HGA-type pectins play crucial roles in mediating the modification of cell wall mechanical properties and controlling turgor-induced plant morphogenesis through the action of pectinolytic enzymes (Levesque-Tremblay et al., 2015; Ali and Traas, 2016). In plants, pectinolytic enzymes or pectinases, which act by hydrolysis of pectic substances through the reactions of depolymerization (hydrolases and lyases) and deesterification (esterases), comprise a heterogeneous group of enzymes, including PMEs, PAEs, PGs, and PLs (Figure 3C). The acetyl- and methyl-esterifications of pectins represent the key parameters for the regulation of cell wall mechanical properties. HGA chains can be deacetylated *in muro* by PAE, with the resulting acetylester change dynamically impacting plant growth and development. It has been demonstrated that the deacetylation of pectin can lower the hydrophobicity of the polysaccharide backbone to increase pectin solubility in water (Rombouts and Thibault, 1986). Thus, PAEs are a crucial structural factor can protect polysaccharides against enzymatic digestion (Liners et al., 1994; Chen and Mort, 1996; Bonnin et al., 2003). Black cottonwood (*Populus trichocarpa*) that overexpress *PtPAE1* exhibit disturbed pollen tube elongation and severe male sterility; however, PtPAE1-mediated deacetylation has been shown to lower the digestibility of pectin (Gou et al., 2012). Following the identification of the Arabidopsis *PAE* family, it was found that *pae8* and *pae9* mutants led to ∼20% increase in acetate accumulation in cell walls leading to the reduction in inflorescence growth (de Souza et al., 2014). Arabidopsis *acetylation 2* (*rwa2*) mutation, which displayed a 20% reduction in cell-wall acetylation, was observed to increased resistance to *Botrytis cinerea* (Manabe et al., 2011). When *Medicago truncatula* was grown in a CO_2_ enriched atmosphere, *PAE* genes were induced in response to aluminum stress and were associated with aluminum resistance (Chandran et al., 2008). In addition, data retrieved from the eFP Browser^1^ showed that Arabidopsis *PAE2* and *PAE4* were induced in response to osmotic and salt stress (Philippe et al., 2017).

Furthermore, the synthesis of HGA with a high methyl ester at C6 carboxyl residues occurs in the Golgi, which is then further exported into the cell wall in a highly methyl-esterified form of 70 ∼ 80% methylesterification (Willats et al., 2001). The action of PME temporally and spatially regulates the fine control of the DM, i.e., the hydrolysis of the methylester bond at the C-6 position of GalA in HGA, and is potentially involved in the regulation of cell wall architecture and determination of the methylesterification status of pectin. The increase of PME activity and DM are attributed to aluminum resistance in the root transition zone in pea (*Pisum sativum*) (Li X. et al., 2016). A limited number of investigations on the patterns of PME action in response to abiotic stresses suggest that this area is largely unknown. Thus, in subsequent discussion we focus on the function of PME to alter cell wall properties through the modification of different wall components, which plays an important role in the response to adverse environments, especially to heat exposure.

### Functions of Pectin Methylesterase

Pectin methylesterases (EC 3.1.1.11), which belong to class 8 (CE8) of the carbohydrate esterases (CAZy website^2^) (Cantarel et al., 2009), and whose activity is regulated by PMEIs, modify the DM of pectins (Pelloux et al., 2007). In the Arabidopsis genome, 66 ORFs have been annotated as putative *PME* genes that are distinctively expressed (Louvet et al., 2006); furthermore, 89 and 80 *PME* ORFs correspond to the protein-coding genes in the poplar (*Populus* spp.) and Asiatic cotton (*Gossypium arboreum*) database, respectively (Geisler-Lee et al., 2006; Li W. et al., 2016). Conversely, fewer *PME* genes, as represented by 43 putative ORFs, were found in rice (*O. sativa* subsp. *Japonica* cv.; Jeong et al., 2015) compared to those of dicots, which may be related to the differences in the structure of the respective cell wall, such as less methyl esterified HGA in grass species (Vogel, 2008; Burton et al., 2010).

**FIGURE 4:**
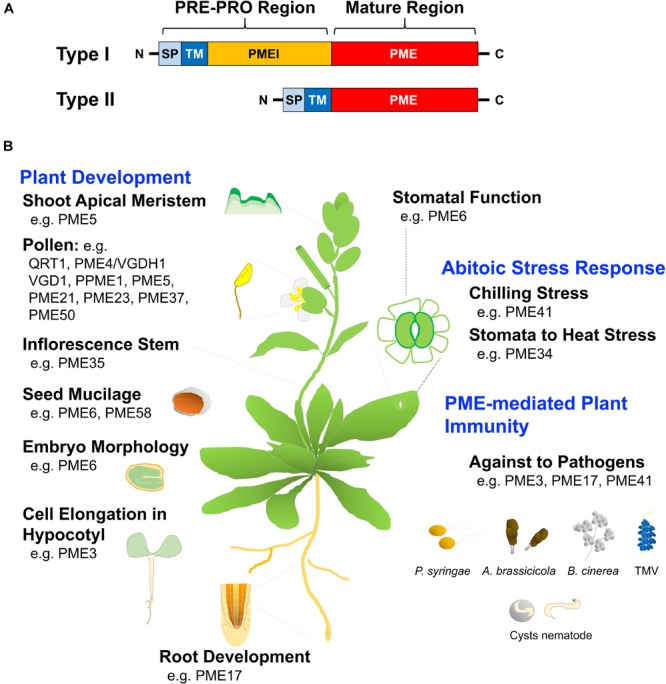
Structural motif and function of pectin methylesterase (PME). **(A)** Types I and II PMEs contain a conserved PME domain, as the active part of the proteins. Type I includes the N-terminal extension of the PRE-PRO region, with the PRE domain containing a signal peptide (SP) or a transmembrane domain (TM) that is required for PME targeting to the cell wall. The N-terminal PRO region shows homology with pectin methylesterase inhibitors (PMEI), whereas type II is characterized by the absence of the PRO region. **(B)** Deficiency in specific *PME* genes reveals multiple roles of PME that have been linked to alteration of plant growth development and the response of plant defenses and abiotic stresses, respectively; related references are indicated in Table 1.

Depending on PME structure, Arabidopsis PMEs are frequently organized with an N-terminal extension of PRE and PRO sequence. PMEs can be classified into types I and II based on their presence or absence of the PRO domain. Type I is characterized by the presence of the N-terminal PRO region, which show homology with PMEI domains, whereas type II is characterized by the absence of the PRO region (Figure 4A). The export of PME to the cell wall via the PRE domain, which can be mediated by a signal peptide or a transmembrane domain (TM or signal anchor), is required for protein targeting (Beigi et al., 2015). The PRO-region is required for correct targeting of the cell wall and supports an autoinhibitory activity of enzymes necessary for secretion of the mature PME to the apoplast (Giovane et al., 2004; Bosch et al., 2005). Type-II PME without the PRO-region and with five or six introns, has a similar structure to that of phytopathogenic organisms, such as fungi and bacteria (Pelloux et al., 2007). The localization of tobacco type-I PME, NtPPME1, was shown using a full-length product fused with GFP that is specifically expressed in the cell wall of pollen, whereas NtPPME1 lacking the PRO-region was maintained in the cytoplasm, suggesting that the PRO-region of NtPPME could assist the correct targeting of the mature PME (Bosch et al., 2005). The TM domain of tobacco PME Q9LEBO assists in the transport of PME to the cell surface and its export to the cell wall; however, the PRO-region of Q9LEBO does not affect targeting to the cell wall (Dorokhov et al., 2006).

### Actions of Pectin Methylesterase

Pectin methylesterases function in de-esterification of the methylated carboxyl group (COOCH_3_) of pectin to form elastic pectins and accompany MeOH generation during division and maturation of the plant cell (Komarova et al., 2014). Three modes of action of mature PMEs on polysaccharides have been proposed: single-chain, multiple-chain, and multiple-attack mechanisms (Aragunde et al., 2018). In the single-chain mechanism, the activity of PME converts all substrate sites on the polymeric chain. In the multiple-chain mechanism, PME catalyzes one reaction and then dissociates from the substrate, whereas PME catalyzes many cycles of reaction before the enzyme-polysaccharide complex dissociates in the multiple-attack mechanism (Beigi et al., 2015; Aragunde et al., 2018). Both single-chain and multiple-attack mechanisms have been proposed in plant and bacterial PMEs as these produce contiguous regions of GalAs (Christensen et al., 1998). Conversely, the random attack of fungal PMEs has been reported to be a multiple-chain mechanism (Duvetter et al., 2006).

During cell wall formation, HGA is de-methylesterified by the activity of PME, which results in contiguous and random patterns of free carboxylic residues. The contiguous demethylesterification of PME (by single-chain or multiple-attack mechanism) leads to large amounts of demethylesterified GalA, the negatively charged chains of which can bind to Ca^2+^ to promote the formation of “egg box” structures and play a significant role in the structural rigidity of the cell wall. Their enzymatic activity can be modulated by different optimal pH values to further shift the mode of action to random demethylesterification (Hocq et al., 2017). Random demethylesterification (as a multiple-chain mechanism) releases protons that become a target for pectin-degrading enzymes such as PG (EC 3.2.1.15), which act by hydrolyzing the α-1,4 link between GalA. PG acts co-operatively with PME to disassemble the pectin polymer networks and contribute to cell wall weakening (Micheli, 2001). This observation has been confirmed by the combination of PME and PG activity causing an increased opening of stomatal aperture in both maize and Asiatic dayflower (*Commelina communis*) (Jones et al., 2005). However, incubation of PG alone did not show the effect on stomata opening, indicating that the methylesterified HGA is crucial for guard cell wall movement (Jones et al., 2005). Under salt stress, the increased demethylesterified pectins mediated by PME activity tend to crosslink with the Ca^2+^, leading to solidification of the cell wall and decreased growth (Uddin et al., 2013). Hence, the degree of pectin methyl-esterification affects Ca^2+^ cross-linking and pectate gel formation, which has dramatic consequences on cell wall texture and mechanical properties, thereby regulating cellular growth, cell shape, and defense reactions in plants (Pelloux et al., 2007).

In addition, the activity of PME is closely regulated by its endogenous inhibitor proteins, PMEIs, during plant development and growth (Micheli, 2001; Giovane et al., 2004). The additional PRO domain in type-I *PME* genes shares similarities with the PMEI domain of *PMEI* genes (Pelloux et al., 2007). PMEIs belong to plant invertase inhibitor-related proteins, and as inhibitors, they play an important role in the regulation of metabolic enzymes (Koch, 1996). A transgenic Arabidopsis that constitutively expresses *AtPMEI-1* or *AtPMEI-2* demonstrates a significant reduction in PME activity and increased levels of pectin methylesterification (Lionetti et al., 2012). Overexpression of a novel *AtPMEI* has a direct, profound effect on the activity of PME. Furthermore, increased PMEI accumulation significantly improved plant resistance to the fungal pathogens *Botrytis cinerea, Bipolaris sorokiniana*, and *Fusarium graminearum* (Lionetti et al., 2007; Volpi et al., 2011). The pepper (*Capsicum annuum*) *CaPMEI* gene, when overexpressed in Arabidopsis, enhances tolerance to *Pseudomonas syringae* pv. tomato, mannitol, and methyl viologen (An et al., 2008). In addition, the overexpression of *PMEI* limits the movement of tobamovirus (tobacco mosaic virus) in tobacco and Arabidopsis, and reduces plant susceptibility to the virus (Lionetti et al., 2014). Arabidopsis *PME3* and *PMEI7* were shown to have overlapping expression patterns in the etiolated hypocotyls when undergoing HGA methylesterification during plant development (Sénéchal et al., 2015). Overexpression of Arabidopsis *PME5* and *PMEI3* resulted in softer and harder shoot apical meristem cell walls, respectively (Peaucelle et al., 2011). Thus, the regulation of *PMEI* genes in the function of PME has a connection with plant development, defense, and stress response including wounding, drought, and oxidative and osmotic stresses (Greiner et al., 1998; An et al., 2008).

### Physiological Roles of Pectin Methylesterase

Pectin methylesterases play an important role in both pectin remodeling and disassembly of the cell wall, and, therefore are involved in many physiological processes, including microsporogenesis, pollen germination, tube growth, pollen separation, seed germination, root development, stem elongation, polarity of leaf growth, and fruit softening during post-harvest fruit ripening (Wen et al., 1999; Pilling et al., 2000; Jiang et al., 2005; Francis et al., 2006; Tian et al., 2006). Moreover, over the past few years, several loss-of-function phenotypes of Arabidopsis *PME* have been described, as shown in Figure 4B and Table 1.

**Table 1:** Identification of specific *Pectin methylesterase* (*PME*) genes reveals multiple roles of *PME* in Arabidopsis.

Genes	AGI code	Functions	Reference
PME3	At3g14310	Cell elongation in hypocotyls. Involved in plant immune responses.	Hewezi et al., 2008
PME5	At5g47500	Involved in shoot apical meristem cell walls.	Peaucelle et al., 2011
PME6	At1g23200	Embryo development. Stomata function.	Levesque-Tremblay et al., 2015; Amsbury et al., 2016
PME7	At1g02810	It may be involved in basal thermotolerance.	Huang et al., 2017
PME17	At2g45220	Involved root development and in response to various stresses.	Sénéchal et al., 2014
PME21	At3g05610	Expressed in dry and imbibed pollen grains.	Mollet et al., 2013; Leroux et al., 2015
PME23	At3g06830	Expressed in dry and imbibed pollen grains.	Mollet et al., 2013
PME34	At3g49220	Involved in thermotolerance.	Huang et al., 2017
PME35	At3g59010	Provides mechanical support to the Arabidopsis stem.	Hongo et al., 2012
PME37	At3g62170	Expressed in dry and imbibed pollen grains.	Mollet et al., 2013; Leroux et al., 2015
PME41	At4g02330	It may serve as one of the mechanisms that BR participates in chilling tolerance of plants.	Qu et al., 2011
PME48	At5g07410	Involved in pollen grain germination.	Mollet et al., 2013; Leroux et al., 2015
PME50	At5g07430	Expressed in dry and imbibed pollen grains.	Mollet et al., 2013; Leroux et al., 2015
PME58	At5g49180	Seed mucilage.	Turbant et al., 2016
PPME1	At1g69940	Promote pollen tube growth. Involved in plant immune responses.	Tian et al., 2006
QUARTET1 (QRT1)	At5g55590	Assisting in the liberation of pollen grains from tetrads during floral development.	Francis et al., 2006
VANGUARD1 (VGD1)	At2g47040	Promote pollen tube growth.	Jiang et al., 2005
VGDH1	At2g47030	VGD1 homolog. Expressed in dry pollen grains.	Jiang et al., 2005; Mollet et al., 2013; Leroux et al., 2015

*QUARTET1* (*QRT1*) assists in the liberation of pollen grains from tetrads during floral development (Francis et al., 2006). *VANGUARD1* (*VGD1*) and *PPME1* (*PME9*) promote pollen tube growth (Jiang et al., 2005; Tian et al., 2006). PME-mediated de-methylesterification is thought to be required to render HGA susceptible to PG-mediated degradation; for example, PME QRT1 potentially acts in tandem with PG QRT3 to degrade de-methylesterified HGA in pollen mother cell primary walls (Rhee et al., 2003; Francis et al., 2006). AtPME35 is responsible for the demethylesterification of pectins and is involved in regulating the mechanical strength of the supporting tissue in Arabidopsis inflorescence stems (Hongo et al., 2012). AtPME6 is abundant during mucilage secretion, acting on embryo morphology and mucilage extrusion, both of which are involved in embryo development (Levesque-Tremblay et al., 2015). In addition, *AtPME58* is specifically expressed in mucilage secretory cells and plays a role in mucilage structure and organization (Turbant et al., 2016). PMEs also act as positive regulators in the control of cell elongation in dark-growth Arabidopsis hypocotyls (Pelletier et al., 2010). *AtPME17* was highly co-expressed with and processed by a subtilisin-type serine protease *AtSBT3.5* to release a mature apoplastic PME isoform that was involved in root development and in response to various stresses (Sénéchal et al., 2014). Pectin content, PME activity, and pectin demethylesterification are also involved in H_2_O_2_-induced cell expansion and in determining the root diameter of rice root tips (Xiong et al., 2015).

Additionally, the DM of HGA settled by PME constitutes an important decisive factor of the biological activity of OG-related signaling and the formation of MeOH, leading to the elicitation of plant defense responses (Osorio et al., 2008). The higher degree of pectin methylesterification is less susceptible to hydrolysis by fungal endo-PG, and, therefore, highly methylesterified pectin can trigger plant resistance to pathogenic fungi (Lionetti et al., 2012). Several studies have reported that PME interaction with a virus-encoded MP is required for tobamovirus, turnip vein clearing virus, and cauliflower mosaic virus infection, mediating cell-to-cell movement of the virus through the plasmodesmata (Chen et al., 2000). AtPME3 interacts with the cellulose binding protein of the cyst nematode *Heterodera schachtii* and enhances the susceptibility of the plant to nematodes (Hewezi et al., 2008). Furthermore, AtPME3 acts as a susceptibility factor and is necessary for the initial colonization by necrotrophic pathogens *B. cinerea* and *Pectobacterium carotovorum* (Raiola et al., 2011). Moreover, PME-mediated pectin methyl de-esterification may influence the mediated release of pectin-derived compounds, which in turn elicits a defense response. Thus, the specific effect of PME in the pattern of pectin methylesterification plays a determinant role in plant immunity (Bethke et al., 2014). Overall, the study of *PME* genes revealed a considerable compatibility and differential control of regulatory pathways in plants.

In addition, some studies have described for the importance of pectin in secondary cell wall formation and modification. Pectin-associated β-1,4-galactans are detected in the secondary walls of tension and compression wood (Mellerowicz and Gorshkova, 2012). The occurrence of the pectin RG-II in the most primitive extant vascular plant groups (e.g., Pteridophytes, Lycophytes, and Bryophytes), is correlated with the upright growth of developed lignified secondary walls in vascular plants (Matsunaga et al., 2004). Additional evidence provided a clearer link between pectin modification and secondary wall formation. The expression of PMEs are involved in the expanding wood cells of poplar (Siedlecka et al., 2008), and in the stem, phloem, and xylem of *Eucalyptus globulus* (Goulao et al., 2011). Arabidopsis mutant lacking *PME35* has been shown reduced the mechanical integrity in their stem interfascicular fibers (Hongo et al., 2012). Hence, pectin plays a role in the early stages of secondary wall deposition and has a fundamental role in secondary wall structure and function (Xiao and Anderson, 2013). However, the ability of cells to adapt to environmental changes through the regulation of PME-mediated modification in secondary cell wall for wall integrity maintenance remains a major challenge.

### Pectin Methyl Esterase Activity in Heat Responses

To date, numerous studies have revealed that PME participates in the regulation of plant development by affecting the mechanical properties of the plant cell walls; however, little is known regarding the role of PME in abiotic stresses. The effects of temperature stress on the cell wall may be revealed at various levels such as cell wall architecture and composition. It has been shown that pectin contents are related to temperature-dependent modifications, and that the DM of pectins is also involved in temperature responses (Solecka et al., 2008; Wu et al., 2010; Lima et al., 2013; Bilska-Kos et al., 2017; Huang et al., 2017).

Available data support the idea that cell wall-modifying enzymes are involved in temperature stress responses. For example, in winter oil-seed rape, the cold temperature-dependent pectin modification through the regulation of pectin methylesterification degree causes a retardation in leaf expansion that is correlated with the development of cold acclimation and fungus resistance (Solecka et al., 2008). In the leaves of chilling-sensitive CM109 maize (*Z. mays* spp. *indentata*, dent), low temperatures of ∼14°C/12°C (day/night) result in a reduction of pectin contents and PME activity, especially after prolonged treatment for 28 h and 7 days (Bilska-Kos et al., 2017). High temperatures of 35–65°C cause an activation of PME activity and the formation of MeOH in the intact tissue of green bean and tomato (Anthon and Barrett, 2006). In winter oilseed rape, HS-induces a nearly 10-fold reduction in *PME35* (EV193389) gene expression (Yu et al., 2014). In tomato pollen, HsfA2 is an important coactivator of HsfA1a during HSR; in addition, in developing anthers of *A2AS* transgenic plants with suppressed *HsfA2* level, approximately 25% of the genes have function codes assigned for cell wall-modifying enzymes (including several *PME, PAE*, and *PL*) under non-stress conditions. It has been suggested that cell wall-related genes might be directly regulated by HsfA2 (Fragkostefanakis et al., 2016). Thus, cell wall-related genes might be regulated by HS-associated gene expression in HSR. The demethylesterification rate of PME activity was increased substantially with increasing temperature, although the mechanism for temperature activation is less understood.

### Pectin Methylesterase Effects on Cellular Calcium Levels

Polysaccharides and pectin present as a Ca^2+^-pectate gel are embedded in the primary-cell-wall matrix, providing an enormous Ca^2+^ reservoir. Pectin contains largely demethylesterified HGA sequences cross-linked through Ca^2+^ bridges to form egg-box structures, which are responsible for maintaining the integrity of the pectic network (Jarvis et al., 2003). The distribution of Ca^2+^ at the cell wall is mainly the result of a plethora of binding sites for Ca^2+^ in the cell wall, as well as the carefully regulated transport of Ca^2+^ into the cytoplasm (Han et al., 2003). Elevated temperature may cause a loss of cell membrane integrity, which allows Ca^2+^ leakage out from the cells into the cell wall to activate PME activity (Anthon and Barrett, 2006). It is possible that, at elevated temperatures, some changes may occur in the PME enzyme that converts it to a different or more active form or that its activity may be increased by the presence of Ca^2+^ and other cations. In previous studies, we verified that fine-tuning of an apoplastic Ca^2+^ mechanism was associated with PME activity on the pectin methylesteri?cation status by immunolocalization analyses of Ca^2+^-demethylated HGA during HSR and EGTA chelator treatment (Wu et al., 2010). The removal of apoplastic Ca^2+^ might participate in HS signaling to induce HS protein expression and cell-wall remodeling to retain plasma membrane integrity, prevent leakage of cellular content and confer thermoprotection (Wu and Jinn, 2010). The blossom-end rot (BER) is a Ca^2+^-related physiological disorder that occurs in tomato fruit. It has been shown that a reduced level of PME expression and activity directly determine a correlation with changes in cellular Ca^2+^ partitioning and distribution in fruits, leading to fruit susceptibility to BER development (de Freitas et al., 2012). The effect of PME expression and activity on the amount of esterified pectins and Ca^2+^ bound to the cell wall is an important factor for plant development and stress responses. Thus, the tight control of the DM of pectin and the formation of Ca^2+^ cross-linkage appears to play a major role in plant growth and act as a regulator in response to heat.

The action of PME and the level of Ca^2+^ availability within the apoplasm has a direct impact on cell wall strength and expansion (Conn et al., 2011). Because the Ca^2+^ binding to uronic acids is easy to exchange for H^+^ (Sentenac and Grignon, 1981), this reaction may be involved in the acid-induced extension of the cell wall. Therefore, the carboxyl groups of pectin likely interact with the charged H^+^ atom that functions to acidify and loosen the cell wall to reduce injury. The cell corners, which contribute to cell adhesion via Ca^2+^ cross-linking, bear greater tension and support the conductivity of mechanical stresses throughout the plant tissue (Ryden et al., 2003). Cleavage of the Ca^2+^ bridges between pectic carboxyl groups in the cell wall is important for cell-wall remodeling during stresses. This suggests that the cell wall regulates the level of Ca^2+^ concentration to make the cell more “relaxed,” thereby increasing the capability to avoid the plasma membrane from detaching from the cell wall. The extra Ca^2+^ is mobilized into the cytoplasm through Ca^2+^ channels that were opened by depolarization. The extracellular influx of Ca^2+^ is governed by changes in the ion binding properties within the cell wall rather than movements across the plasma membrane (Holdaway-Clarke et al., 1997). Moreover, pectin gel strength increases with increasing Ca^2+^ concentration but decreases with increased temperature and acidity (Lootens et al., 2003). Thus, the cell wall needs to eliminate Ca^2+^ and maintain low-level apoplastic Ca^2+^ during HS, resulting in increasing Ca^2+^ levels in the cytoplasm for regulating intracellular levels in response to HS (Wu et al., 2010).

Because MeOH is a product of PME action, it might serve as a volatile signal in the protection of photosynthetic machinery from photo-inhibition; stimulating the growth of C3 plants and the signaling of plant-herbivore interactions for plant defense mechanisms (Nonomura and Benson, 1992; Frenkel et al., 1998; Von Dahl et al., 2006). Furthermore, MeOH activates various patterns of gene expression that are involved in detoxification and signaling pathways, including the induction of *HSP* genes (Downie et al., 2004). The OGs, as pectin fragments related to PME activities that act as elicitors to stimulate the production of ROS, plasma membrane depolarization, and increased inositol triphosphate and [Ca^2+^]_cyt_, have been widely reported in plants (Moscatiello et al., 2006). It has been shown that the extracellular domain of WAK1, which functions as a potential sensor of cell wall signaling by directly binding to the Ca^2+^ crosslinking pectin-derived OGs, is involved in cell growth, cell expansion, and disease resistance (Wagner and Kohorn, 2001; Decreux and Messiaen, 2005; Kohorn et al., 2006; Li et al., 2009). The heat-activated PME participates in pectin remodeling, which in turn keeps cells from separating and maintains plasma-membrane integrity, prevents cellular leakage, and coordinates with HS signaling to confer thermoprotection (Wu and Jinn, 2010). Together, these findings suggest that homeostasis of the apoplastic [Ca^2+^] through the regulation of PME activity during HSR might have a pronounced effect on the development of heat tolerance by preventing cellular leakage through Ca^2+^-pectate remodeling in the cell wall.

### Guard Cell Wall Remodeling in Heat Responses

**FIGURE 5:**
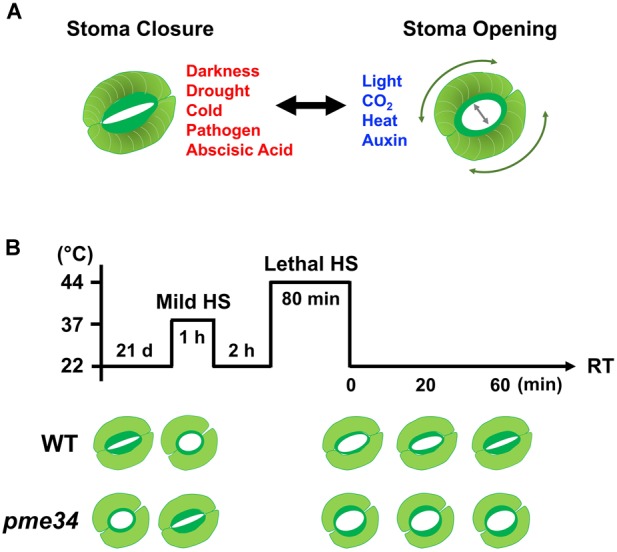
Arabidopsis PME34 regulates the stomatal aperture under heat stress. **(A)** The inner wall of a guard cell is thicker and more elastic than the outer cell wall to facilitate the opening of the stomatal pore. The elastic property of the guard cell wall acts reversibly during stomatal opening and closing owing to differential thickening and the orientation of cellulose microfibrils (expressed in threads). The openings and closures of the stomata pore are strictly regulated by the integration of environmental stimuli and endogenous hormonal signals. **(B)** Comparison of elevated temperature stimulated-stomatal opening in wild-type (WT; Col) and *pme34* mutant plants. Leaves (21-day-old) of WT and *pme34* plants were treated with 37°C-mild and 44°C-lethal heat stress (LHS), respectively, as indicated. The pictogram shows the HS regime and the schematic diagram in the lower panel indicates the response of the stomatal aperture. Under normal condition, *pme34* plants had a larger stomata aperture compared with that of WT plants. Under mild-HS treatment, the stomatal apertures in WT plants increased for aiding the dissipation of heat, whereas those of *pme34* plants did not. Following the further 44°C LHS at recovery time (RT), stomatal apertures of *pme34* were opened wider than those of WT plants, indicating greater water loss than that in WT plants.

Guard cells comprise a highly developed system that is used to determine and characterize the mechanism of the early signal transduction pathway in plants. In particular, they are involved in gas exchange between the interior of the plant and the external environment through the regulation of successive openings and closures of the stomatal pore. Guard cells perceive a multitude of endogenous and environmental stimuli including hormonal stimuli, light, humidity, CO_2_ concentration, drought, and temperature to trigger cellular responses resulting in stomatal opening or closure (Kim et al., 2010; Wu et al., 2017). High temperature increases the risk of heat damage and water shortage to plants. In response to elevated temperatures, transpiration occurs through the opening of stomatal apertures to facilitate cooling of the leaf surface through water evaporation (Figure 5A). In contrast, drought can cause stomatal closure and reduce transpiration rates; therefore, stomatal control is considered to be a short-term dynamic adaptation to avoid the reduction in leaf water potential (Osakabe et al., 2014).

Immunolocalization analyses of Arabidopsis leaf sections indicate highly methylesterified and Ca^2+^ cross-linked de-esterified HGA in mesophyll cells, whereas unesterified HGA constitutes the predominant form of pectin in guard-cell walls, leading the stomatal closure response (Amsbury et al., 2016). Arabidopsis *PME6* and *polygalacturonase involved in expansion 3* (*PGX3*) in the guard cells play an important role in response to stomatal opening/closure control (Amsbury et al., 2016; Rui et al., 2017). In a previous study, heat-exposed rice plants exhibited lower stomatal conductance until harvest, which can affect carbon balance, grain-filling processes, and yield production (Yang and Heilman, 1991). In *C. communis*, it has been shown that a 40°C HS for 5 min in roots could lead to a significant decrease in stomatal conductance, indicating that the communication between root and shoot is mediated by long-distance signaling (Yang S. et al., 2006). In *Mimosa pudica*, heat stimulation triggers rapid hydro-passive stomatal opening and subsequent stomatal closure that is concomitant with a loss of net CO_2_ uptake (Kaiser and Grams, 2006).

The highly specialized walls of guard cells enable them to undergo large and reversible deformation during the constriction of stomata (Wu et al., 2017). Therefore, it is possible that cell wall modification factors are involved in controlling stomata apertures. Recently, we found that Arabidopsis *PME34* deficiency causes lower transpiration rates owing to an abnormal stomatal opening, leading to higher leaf temperatures and enhanced sensitivity to heat (Huang et al., 2017). The type-I *PME* gene *PME34*, which encodes a plasma membrane-localized and a cell wall deposited protein, functions during guard cell wall modification in response to heat. *PME34* mutants have been shown to be hypersensitive to heat but independent of HSF-mediated *HSP* gene transcriptional activation. The *PME34* transcript was induced by ABA and highly expressed in guard cells, indicating that PME34 is associated with ABA-dependent stomatal movement in response to heat (Huang et al., 2017). High PME activity coincided with an increase in PG activity in *pme34* plants, degrading pectin more easily, and further influencing the ability of guard cell walls to be modified in response to heat. This may support the idea proposed by Wu and Jinn (2010), who suggested that different PME isoforms exhibit distinct action patterns and pectic substrate specificity in response to HS. As highly methylesterified pectins are less susceptible to the action of PG, HS might render the cell wall to be more acidic so that it could stimulate the random demethylesterification activity of PME and promote the action of PG on pectin cleavage, further influencing the structural characteristics of guard cell walls for stomatal movement. Notably, the absence of *PME34* activity in guard cells may be complemented by other PMEs and an integration with PG action to bring about the wide opening of stomata pores (Figure 5B).

Thus, PME34 may have a role in crosslinking with pectic polymers in the cell wall to regulate the flexibility of guard cell walls (Huang et al., 2017; Wu et al., 2017). Although PME functions to remove the methylester group from HGA to prevent stomatal opening, loss of *PME34* resulted in wider stomata under lethal heat treatment. This is consistent with the observation that during drought stress, *pme6* mutants have a significantly cooler leaf temperature than the wild-type plants, as well as a more restricted response to ABA (Amsbury et al., 2016). The *pme34* mutant displayed a defect in the control of stomatal movement with a concomitant increase in leaf temperature. It also showed a higher transpiration rate through the more widely open stomata, which was probably due to the altered pectin methylesterification status of the guard cell wall properties. Thus, *PME34* functions in controlling stomatal movements and in regulating the flexibility of the guard cell wall, which is required for the heat response. The impact of loss of *PME34* on stomatal aperture may be due to Ca^2+^ signaling or oligosaccharides released during cell-wall modification, or both, which requires further investigation (Wu et al., 2017).

## Conclusion and Prospective

Although fine-tuning of the methylesterification of pectin through the regulation of PME activity during plant growth is relatively well understood, very little is known about stress-induced alterations of cell-wall polymers with respect to PME activity. Analysis of the patterns of pectin methylesterification in *pme* mutants is important to distinguish the distinct roles of individual *PME* genes. The evidence from genetic and transgenic plants indicated that the modification of cell wall remodeling has a pronounced effect on stress tolerance. The adjustment of the cell wall through the activity of PME under abiotic stresses is a critical determinant of plant adaptation. The change in cell wall metabolism and cell wall-modifying enzyme activity in controlling cell wall plasticity is an important physiological mechanism of plants in response to heat. The stress effect on the architecture of cell wall remodeling by PME activity may depend on the plant species, genotype, and growth stage, and also rely on the intensity and timing of the stress. In addition, the specificity of PMEI toward different PME isoforms can directly modulate the endogenous PME activity during plant development and various stress responses. In particular, complex interaction between PMEs and their inhibitors appears to be involved in a complex metabolic network and the regulation of gene expression pathways during plant growth and development as well as in stress adaptation. The additional complexity of the interaction of PME with other cell-wall proteins to render a load-bearing, yet extensible primary cell wall during stress, remains an elusive issue. Much remains to be elucidated as to how the cell wall senses and transduces the signals leading to stress-induced transcriptional machinery changes and the underlying cell-wall polysaccharide deposition and modification. The role of cell wall-related genes, such as WAKs, which directly bind pectin polymers and partially depend upon the DM of pectin, and polysaccharides, has been explored during various stages of plant development (Kohorn et al., 2009; Tucker et al., 2018). The qualitative and quantitative assessment of cell wall composition at the single cell level is also required (Tucker et al., 2018). In particular, we need to elucidate single-cell responses to certain environmental changes. For instance, in the root cells of Arabidopsis, transcriptional changes were found to be directly related to alternations of cell-wall composition (Somssich et al., 2016), indicating that transcript abundance is followed by associated cell-wall modifying enzymes and proteins. Further, it is required to establish a direct connection between pectin modification and secondary wall formation by identifying and determining the function of pectin-related genes. Consequently, PME-mediated deesterification could be a crucial mechanism for contributing the secondary wall growth of wood development. Likewise, the transcriptional regulation of pectin-modifying genes might be an important aspect of secondary cell wall formation attributed to both abiotic and microbial challenges. These insights provide a foundation for further research such as transcriptomics studies that may reveal details of the cell wall machinery and stress-responsive transcription factors to provide targets and strategies to facilitate plant adaptation to HS.

## Author Contributions

H-CW and T-LJ conceived and wrote the manuscript. VB contributed to the final version of the manuscript.

## Conflict of Interest Statement

The authors declare that the research was conducted in the absence of any commercial or financial relationships that could be construed as a potential conflict of interest.

## Footnotes

1 http://bar.utoronto.ca/efp/cgi-bin/efpWeb.cgi

2 http://www.cazy.org

## Abbreviations

ABA: abscisic acid
AGA: apiogalacturonan
Ca^2+^: calcium ion
CaM: calmodulin
CBK3: CaM-binding protein kinase 3
CesA: cellulose synthase
CSLD5: cellulose synthase-like protein
CTL1: chitinase-like protein
DM: degree of methylesterification
EXP: expansin
GalA: galacturonic acid
HGA: homogalacturonan
HIT4: heat-intolerant 4
HS: heat stress
HSBP: HSF-binding protein
HSE: heat shock element
HSF: heat shock transcription factor
HSP: heat stress protein
HSR: heat stress response
MeOH: methanol
miRNA: microRNA
MP: movement protein
NF-YC10: nuclear factor Y, subunit C10
NPG1: no pollen germination 1
OG: oligogalacturonide
ORF: open reading frame
PAE: pectin acetylesterase
PG: polygalacturonase
PL: pectin lyase
PLL: pectate lyase-like protein
PME: pectin methylesterase
PME34: pectin methylesterase 34
PMEI: PME inhibitor
QRT1: QUARTET 1
RG-I: rhamnogalacturonan-I
RG-II: rhamnogalacturonan-II
ROS: reactive oxygen species
sHSP: small heat shock protein
siRNA: small interfering RNA
VGD1: VANGUARD 1
WAK: wall-associated kinase
XET/XTH: xyloglucan endotransglycosylase/hydrolase
XGA: xylogalacturonan

## References

[B1] Ali O. Traas J. (2016). Force-driven polymerization and turgor-induced wall expansion. *Trends Plant Sci.* 21 398–409. 10.1016/j.tplants.2016.01.019 26895732

[B2] Amsbury S. Hunt L. Elhaddad N. Baillie A. Lundgren M. Verhertbruggen Y. (2016). Stomatal function requires pectin de-methyl-esterification of the guard cell wall. *Curr. Biol.* 26 2899–2906. 10.1016/j.cub.2016.08.021 27720618 PMC5106435

[B3] An S. H. Sohn K. H. Choi H. W. Hwang I. S. Lee S. C. Hwang B. K. (2008). Pepper pectin methylesterase inhibitor protein CaPMEI1 is required for antifungal activity, basal disease resistance and abiotic stress tolerance. *Planta* 228 61–78. 10.1007/s00425-008-0719-z 18327607 PMC2413075

[B4] Anthon G. E. Barrett D. M. (2006). Characterization of the temperature activation of pectin methylesterase in green beans and tomatoes. *J. Agric. Food Chem.* 54 204–211. 10.1021/jf051877q 16390200

[B5] Aragunde H. Biarnés X. Planas A. (2018). Substrate recognition and specificity of chitin deacetylases and related family 4 carbohydrate esterases. *Int. J. Mol. Sci.* 19:412. 10.3390/ijms19020412 29385775 PMC5855634

[B6] Ballén-Taborda C. Plata G. Ayling S. Rodríguez-Zapata F. Lopez-Lavalle L. A. B. Duitama J. (2013). Identification of cassava microRNAs under abiotic stress. *Int. J. Genomics* 2013:857986. 10.1155/2013/857986 24328029 PMC3845235

[B7] Bäurle I. (2016). Plant heat adaptation: priming in response to heat stress. *F1000Res.* 5:694. 10.12688/f1000research.7526.1 27134736 PMC4837978

[B8] Beigi T. M. Ngadi M. O. Holman D. B. Chénier M. R. (2015). Pectin methylesterases: a review. *J. Bioprocess. Biotech.* 5:227.

[B9] Bethke G. Grundman R. E. Sreekanta S. Truman W. Katagiri F. Glazebrook J. (2014). *Arabidopsis* pectin methylesterases contribute to immunity against *Pseudomonas syringae*. *Plant Physiol.* 164 1093–1107. 10.1104/pp.113.227637 24367018 PMC3912082

[B10] Bharti K. Von Koskull-Doring P. Bharti S. Kumar P. Tintschl-Korbitzer A. Treuter E. (2004). Tomato heat stress transcription factor HsfB1 represents a novel type of general transcription coactivator with a histone-like motif interacting with the plant CREB binding protein ortholog HAC1. *Plant Cell* 16 1521–1535. 10.1105/tpc.019927 15131252 PMC490043

[B11] Bilska-Kos A. Solecka D. Dziewulska A. Ochodzki P. Jończyk M. Bilski H. (2017). Low temperature caused modifications in the arrangement of cell wall pectins due to changes of osmotic potential of cells of maize leaves (*Zea mays* L.). *Protoplasma* 254 713–724. 10.1007/s00709-016-0982-y 27193139 PMC5309300

[B12] Bonnin E. Le Goff A. Van Alebeek G. W. M. Voragen A. G. J. Thibault J. F. (2003). Mode of action of *Fusarium moniliforme* endopolygalacturonase towards acetylated pectin. *Carbohydr. Polym.* 52 381–388. 10.1016/S0144-8617(02)00332-6 18852070

[B13] Bosch M. Cheung A. Y. Hepler P. K. (2005). Pectin methylesterase, a regulator of pollen tube growth. *Plant Physiol.* 138 1334–1346. 10.1104/pp.105.059865 15951488 PMC1176407

[B14] Boyko A. Blevins T. Yao Y. Golubov A. Bilichak A. Ilnytskyy Y. (2010). Transgenerational adaptation of *Arabidopsis* to stress requires DNA methylation and the function of dicer-like proteins. *PLoS One* 5:e9514. 10.1371/journal.pone.0009514 20209086 PMC2831073

[B15] Braam J. Davis R. W. (1990). Rain-, wind-, and touch-induced expression of calmodulin and calmodulin-related genes in *Arabidopsis*. *Cell* 60 357–364. 10.1016/0092-8674(90)90587-5 2302732

[B16] Burton R. A. Gidley M. J. Fincher G. B. (2010). Heterogeneity in the chemistry, structure and function of plant cell walls. *Nat. Chem. Biol.* 6:724. 10.1038/nchembio.439 20852610

[B17] Busch W. Wunderlich M. Schöffl F. (2005). Identification of novel heat shock factor-dependent genes and biochemical pathways in *Arabidopsis thaliana*. *Plant J.* 41 1–14. 10.1111/j.1365-313X.2004.02272.x 15610345

[B18] Caffall K. H. Mohnen D. (2009). The structure, function, and biosynthesis of plant cell wall pectic polysaccharides. *Carbohyd. Res.* 344 1879–1900. 10.1016/j.carres.2009.05.021 19616198

[B19] Cantarel B. L. Coutinho P. M. Rancurel C. Bernard T. Lombard V. Henrissat B. (2009). The carbohydrate-active EnZymes database (CAZy): an expert resource for glycogenomics. *Nucleic Acids Res.* 37 D233–D238. 10.1093/nar/gkn663 18838391 PMC2686590

[B20] Carvalho C. P. Hayashi A. H. Braga M. R. Nievola C. C. (2013). Biochemical and anatomical responses related to the in vitro survival of the tropical bromeliad *Nidularium minutum* to low temperatures. *Plant Physiol. Biochem.* 71 144–154. 10.1016/j.plaphy.2013.07.005 23917072

[B21] Cavrak V. V. Lettner N. Jamge S. Kosarewicz A. Bayer L. M. Mittelsten Scheid O. (2014). How a retrotransposon exploits the plant’s heat stress response for its activation. *PLoS Genet.* 10:e1004115. 10.1371/journal.pgen.1004115 24497839 PMC3907296

[B22] Cha J. Y. Su’udi M. Kim W. Y. Kim D. R. Kwak Y. S. Son D. (2012). Functional characterization of orchardgrass cytosolic Hsp70 (DgHsp70) and the negative regulation by Ca2+/AtCaM2 binding. *Plant Physiol. Biochem.* 58 29–36. 10.1016/j.plaphy.2012.06.006 22771433

[B23] Chandran D. Sharopova N. Ivashuta S. Gantt J. S. VandenBosch K. A. Samac D. A. (2008). Transcriptome profiling identified novel genes associated with aluminum toxicity, resistance and tolerance in *Medicago truncatula*. *Planta* 228 151–166. 10.1007/s00425-008-0726-0 18351384

[B24] Charng Y. Y. Liu H. C. Liu N. Y. Chi W. T. Wang C. N. Chang S. H. (2007). A heat-inducible transcription factor, HsfA2 is required for extension of acquired thermotolerance in *Arabidopsis. Plant Physiol.* 143 251–262. 10.1104/pp.106.091322 17085506 PMC1761974

[B25] Charng Y. Y. Liu H. C. Liu N. Y. Hsu F. C. Ko S. S. (2006). *Arabidopsis* Hsa32, a novel heat shock protein, is essential for acquired thermotolerance during long recovery after acclimation. *Plant Physiol.* 140 1297–1305. 10.1104/pp.105.074898 16500991 PMC1435801

[B26] Chen E. M. W. Mort A. J. (1996). Nature of sites hydrolyzable by endopolygalacturonase in partially-esterified homogalacturonans. *Carbohydr. Polym.* 29 129–136. 10.1016/0144-8617(96)00005-7

[B27] Chen M. H. Sheng J. Hind G. Handa A. K. Citovsky V. (2000). Interaction between the tobacco mosaic virus movement protein and host cell pectin methylesterases is required for viral cell-to-cell movement. *EMBO J.* 19 913–920. 10.1093/emboj/19.5.913 10698933 PMC305631

[B28] Chen Z. Hong X. I. Zhang H. Wang Y. Li X. Zhu J. K. (2005). Disruption of the cellulose synthase gene, AtCesA8/IRX1, enhances drought and osmotic stress tolerance in *Arabidopsis*. *Plant J.* 43 273–283. 10.1111/j.1365-313X.2005.02452.x 15998313

[B29] Choi J. Y. Seo Y. S. Kim S. J. Kim W. T. Shin J. S. (2011). Constitutive expression of CaXTH3, a hot pepper xyloglucan endotransglucosylase/hydrolase, enhanced tolerance to salt and drought stresses without phenotypic defects in tomato plants (*Solanum lycopersicum* cv, Dotaerang). *Plant Cell Rep.* 30 867–877. 21207033 10.1007/s00299-010-0989-3

[B30] Christensen T. M. Nielsen J. E. Kreiberg J. D. Rasmussen P. Mikkelsen J. D. (1998). Pectin methyl esterase from orange fruit: characterization and localization by in-situ hybridization and immunohistochemistry. *Planta* 206 493–503. 10.1007/s004250050426 9821684

[B31] Coenen G. J. Bakx E. J. Verhoef R. P. Schols H. A. Voragen A. G. J. (2007). Identification of the connecting linkage between homo- or xylogalacturonan and rhamnogalacturonan type I. *Carbohydr. Polym.* 70 224–235. 10.1016/j.carbpol.2007.04.007

[B32] Conn S. J. Gilliham M. Athman A. Schreiber A. W. Baumann U. Moller I. (2011). Cell-specific vacuolar calcium storage mediated by CAX1 regulates apoplastic calcium concentration, gas exchange, and plant productivity in *Arabidopsis*. *Plant Cell* 23 240–257. 10.1105/tpc.109.072769 21258004 PMC3051233

[B33] Corcoran E. E. Means A. R. (2001). Defining Ca2+/calmodulin-dependent protein kinase cascades in transcriptional regulation. *J. Biol. Chem.* 276 2975–2978. 10.1074/jbc.R000027200 11096122

[B34] Corneliussen B. Holm M. Waltersson Y. Onions J. Hallberg B. Thornell A. (1994). Calcium/calmodulin inhibition of basic-helix-loop-helix transcription factor domains. *Nature* 368:760. 10.1038/368760a0 8152489

[B35] Cosgrove D. J. (2015). Plant expansins: diversity and interactions with plant cell walls. *Curr. Opin. Plant Boil* 25 162–172. 10.1016/j.pbi.2015.05.014 26057089 PMC4532548

[B36] de Freitas S. T. Handa A. K. Wu Q. Park S. Mitcham E. J. (2012). Role of pectin methylesterases in cellular calcium distribution and blossom-end rot development in tomato fruit. *Plant J.* 71 824–835. 10.1111/j.1365-313X.2012.05034.x 22563738

[B37] de Souza A. Hull P. A. Gille S. Pauly M. (2014). Identification and functional characterization of the distinct plant pectin esterases PAE8 and PAE9 and their deletion mutants. *Planta* 240 1123–1138. 10.1007/s00425-014-2139-6 25115560 PMC4200376

[B38] Decreux A. Messiaen J. (2005). Wall-associated kinase WAK1 interacts with cell wall pectins in a calcium-induced conformation. *Plant Cell Physiol.* 46 268–278. 10.1093/pcp/pci026 15769808

[B39] Demarty M. Morvan C. Thellier M. (1984). Calcium and the cell wall. *Plant Cell Environ.* 7 441–448. 10.1111/j.1365-3040.1984.tb01434.x

[B40] Demidchik V. Maathuis F. J. M. (2007). Physiological roles of nonselective cation channels in plants: from salt stress to signalling and development. *New Phytol.* 175 387–404. 10.1111/j.1469-8137.2007.02128.x 17635215

[B41] Dodd A. N. Kudla J. Sanders D. (2010). The language of calcium signaling. *Annu. Rev. Plant Biol.* 61 593–620. 10.1146/annurev-arplant-070109-104628 20192754

[B42] Dolmetsch R. E. Lewis R. S. Goodnow C. C. Healy J. I. (1997). Differential activation of transcription factors induced by Ca2+ response amplitude and duration. *Nature* 386:855. 10.1038/386855a0 9126747

[B43] Dorokhov Y. L. Skurat E. V. Frolova O. Y. Gasanova T. V. Ivanov P. A. Ravin N. V. (2006). Role of the leader sequence in tobacco pectin methylesterase secretion. *FEBS Lett.* 580 3329–3334. 10.1016/j.febslet.2006.04.090 16709413

[B44] Downie A. Miyazaki S. Bohnert H. John P. Coleman J. Parry M. (2004). Expression profiling of the response of *Arabidopsis thaliana* to methanol stimulation. *Phytochemistry* 65 2305–2316. 10.1016/j.phytochem.2004.07.006 15381001

[B45] Duvetter T. Fraeye I. Sila D. Verlent I. Smout C. Hendrickx M. (2006). Mode of de-esterification of alkaline and acidic pectin methyl esterases at different pH conditions. *J. Agric. Food Chem.* 54 7825–7831. 10.1021/jf060013h 17002458

[B46] Edreva A. Sotirova V. Georgieva I. D. Stoimenova E. Rodeva R. Bogatzevska N. (2000). Differential expression of β-glucosidase in tomato-stress stimuli systems. *Acta Physiol. Plant.* 22 274–277. 10.1007/s11738-000-0031-4

[B47] Folsom J. J. Begcy K. Hao X. Wang D. Walia H. (2014). Rice fertilization-independent endosperm1 regulates seed size under heat stress by controlling early endosperm development. *Plant Physiol.* 165 238–248. 10.1104/pp.113.232413 24590858 PMC4012583

[B48] Fragkostefanakis S. Mesihovic A. Simm S. Paupière M. J. Hu Y. Paul P. (2016). HsfA2 controls the activity of developmentally and stress-regulated heat stress protection mechanisms in tomato male reproductive tissues. *Plant Physiol.* 170 2461–2477. 10.1104/pp.15.01913 26917685 PMC4825147

[B49] Francis K. E. Lam S. Y. Copenhaver G. P. (2006). Separation of *Arabidopsis* pollen tetrads is regulated by QUARTET1, a pectin methylesterase gene. *Plant Physiol.* 142 1004–1013. 10.1104/pp.106.085274 16980565 PMC1630721

[B50] Frenkel C. Peters J. S. Tieman D. M. Tiznado M. E. Handa A. K. (1998). Pectin methylesterase regulates methanol and ethanol accumulation in ripening tomato (*Lycopersicon esculentum*) fruit. *J. Biol. Chem.* 273 4293–4295. 10.1074/jbc.273.8.4293 9468474

[B51] Fu S. Meeley R. Scanlon M. J. (2002). *Empty pericarp2* encodes a negative regulator of the heat shock response and is required for maize embryogenesis. *Plant Cell* 14 3119–3132. 10.1105/tpc.006726 12468731 PMC151206

[B52] Geisler-Lee J. Geisler M. Coutinho P. M. Segerman B. Nishikubo N. Takahashi J. (2006). Poplar carbohydrate-active enzymes, gene identification and expression analyses. *Plant Physiol.* 140 946–962. 10.1104/pp.105.072652 16415215 PMC1400564

[B53] Giovane A. Servillo L. Balestrieri C. Raiola A. D’avino R. Tamburrini M. (2004). Pectin methylesterase inhibitor. *Biochim. Biophys. Acta* 1696 245–252. 10.1016/j.bbapap.2003.08.011 14871665

[B54] Gong M. Van Der Luit A. H. Knight M. R. Trewavas A. J. (1998). Heat-shock-induced changes in intracellular Ca2+ level in tobacco seedlings in relation to thermotolerance. *Plant Physiol.* 116 429–437. 10.1104/pp.116.1.429

[B55] Gou J. Y. Miller L. M. Hou G. Yu X. H. Chen X. Y. Liu C. J. (2012). Acetylesterase-mediated deacetylation of pectin impairs cell elongation, pollen germination, and plant reproduction. *Plant Cell* 24 50–65. 10.1105/tpc.111.092411 22247250 PMC3289554

[B56] Goulao L. F. Vieira-Silva S. Jackson P. A. (2011). Association of hemicellulose- and pectin-modifying gene expression with *Eucalyptus globulus* secondary growth. *Plant Physiol. Biochem.* 49 873–881. 10.1016/j.plaphy.2011.02.020 21429757

[B57] Greiner S. Krausgrill S. Rausch T. (1998). Cloning of a tobacco apoplasmic invertase inhibitor: proof of function of the recombinant protein and expression analysis during plant development. *Plant Physiol.* 116 733–742. 10.1104/pp.116.2.733 9489020 PMC35133

[B58] Hahn A. Bublak D. Schleiff E. Scharf K. D. (2011). Crosstalk between Hsp90 and Hsp70 chaperones and heat stress transcription factors in tomato. *Plant Cell* 23 741–755. 10.1105/tpc.110.076018 21307284 PMC3077788

[B59] Han S. Tang R. Anderson L. K. Woerner T. E. Pei Z. M. (2003). A cell surface receptor mediates extracellular Ca2+ sensing in guard cells. *Nature* 425:196. 10.1038/nature01932 12968184

[B60] Harholt J. Suttangkakul A. Vibe Scheller H. (2010). Biosynthesis of pectin. *Plant Physiol.* 153 384–395. 10.1104/pp.110.156588 20427466 PMC2879803

[B61] Hewezi T. Howe P. Maier T. R. Hussey R. S. Mitchum M. G. Davis E. L. (2008). Cellulose binding protein from the parasitic nematode *Heterodera schachtii* interacts with *Arabidopsis* pectin methylesterase: cooperative cell wall modification during parasitism. *Plant Cell* 20 3080–3093. 10.1105/tpc.108.063065 19001564 PMC2613657

[B62] Hirschi K. D. (2004). The calcium conundrum, both versatile nutrient and specific signal. *Plant Physiol.* 136 2438–2442. 10.1104/pp.104.046490 15375199 PMC523310

[B63] Hocq L. Pelloux J. Lefebvre V. (2017). Connecting homogalacturonan-type pectin remodeling to acid growth. *Trends Plant Sci.* 22 20–29. 10.1016/j.tplants.2016.10.009 27884541

[B64] Holdaway-Clarke T. L. Feijo J. A. Hackett G. R. Kunkel J. G. Hepler P. K. (1997). Pollen tube growth and the intracellular cytosolic calcium gradient oscillate in phase while extracellular calcium influx is delayed. *Plant Cell* 9 1999–2010. 10.1105/tpc.9.11.1999 12237353 PMC157053

[B65] Hongo S. Sato K. Yokoyama R. Nishitani K. (2012). Demethylesterification of the primary wall by PECTIN METHYLESTERASE35 provides mechanical support to the *Arabidopsis* stem. *Plant Cell* 24 2624–2634. 10.1105/tpc.112.099325 22693281 PMC3406921

[B66] Houston K. Tucker M. R. Chowdhury J. Shirley N. Little (2016). The plant cell wall: a complex and dynamic structure as revealed by the responses of genes under stress conditions. *Front. Plant Sci.* 7:984. 10.3389/fpls.2016.00984 27559336 PMC4978735

[B67] Hsu S. F. Lai H. C. Jinn T. L. (2010). Cytosol-localized heat shock factor-binding protein, AtHSBP, functions as a negative regulator of heat shock response by translocation to the nucleus and is required for seed development in *Arabidopsis*. *Plant Physiol.* 153 773–784. 10.1104/pp.109.151225 20388662 PMC2879799

[B68] Huang Y. C. Niu C. Y. Yang C. R. Jinn T. L. (2016). The heat stress factor HSFA6b connects ABA signaling and ABA-mediated heat responses. *Plant Physiol.* 172 1182–1199. 10.1104/pp.16.00860 27493213 PMC5047099

[B69] Huang Y. C. Wu H. C. Wang Y. D. Liu C. H. Lin C. C. Luo D. L. (2017). PECTIN METHYLESTERASE34 contributes to heat tolerance through its role in promoting stomatal movement. *Plant Physiol.* 174 748–763. 10.1104/pp.17.00335 28381503 PMC5462046

[B70] Ikeda M. Mitsuda N. Ohme-Takagi M. (2011). *Arabidopsis* HsfB1 and HsfB2b act as repressors of the expression of heat-inducible Hsfs but positively regulate the acquired thermotolerance. *Plant Physiol.* 157 1243–1254. 10.1104/pp.111.179036 21908690 PMC3252156

[B71] Imazu H. Sakurai H. (2005). *Saccharomyces cerevisiae* heat shock transcription factor regulates cell wall remodeling in response to heat shock. *Eukaryot. Cell* 4 1050–1056. 10.1128/EC.4.6.1050-1056.2005 15947197 PMC1151985

[B72] IPCC. (2012). “Managing the risks of extreme events and disasters to advance climate change adaptation,” in *A Special Report of Working Groups I and Ii of the Intergovernmental Panel on Climate Change* eds Field C. B Barros V. Stocker T. F. Qin D. Dokken D. J. Ebi K. L. (Cambridge: Cambridge University Press). 10.1136/jech-2012-201045 22766781

[B73] İşkil R. Surgun-Acar Y. (2018). Expression analysis of cell wall assembly and remodelling-related genes in *Arabidopsis* roots subjected to boron stress and brassinosteroid at different developmental stages. *Acta Bot. Bras.* 32 546–554. 10.1590/0102-33062018abb0023

[B74] Ito H. Gaubert H. Bucher E. Mirouze M. Vaillant I. Paszkowski J. (2011). An siRNA pathway prevents transgenerational retrotransposition in plants subjected to stress. *Nature* 472 115–119. 10.1038/nature09861 21399627

[B75] Jarvis M. C. Briggs S. P. H. Knox J. P. (2003). Intercellular adhesion and cell separation in plants. *Plant Cell Environ.* 26 977–989. 10.1046/j.1365-3040.2003.01034.x

[B76] Jeong H. Y. Nguyen H. P. Lee C. (2015). Genome-wide identification and expression analysis of rice pectin methylesterases: implication of functional roles of pectin modification in rice physiology. *J. Plant Physiol.* 183 23–29. 10.1016/j.jplph.2015.05.001 26072144

[B77] Jiang L. Yang S. L. Xie L. F. Puah C. S. Zhang X. Q. Yang W. C. (2005). VANGUARD1 encodes a pectin methylesterase that enhances pollen tube growth in the *Arabidopsis* style and transmitting tract. *Plant Cell* 17 584–596. 10.1105/tpc.104.027631 15659637 PMC548828

[B78] Jones L. Milne J. L. Ashford D. Mccann M. C. Mcqueen-Mason S. J. (2005). A conserved functional role of pectic polymers in stomatal guard cells from a range of plant species. *Planta* 221 255–264. 10.1007/s00425-004-1432-1 15578215

[B79] Kaiser H. Grams T. E. E. (2006). Rapid hydropassive opening and subsequent active stomatal closure follow heat-induced electrical signals in *Mimosa pudica*. *J. Exp. Bot.* 57 2087–2092. 10.1093/jxb/erj165 16698819

[B80] Khraiwesh B. Zhu J. K. Zhu J. (2012). Role of miRNAs and siRNAs in biotic and abiotic stress responses of plants. *Biochim. Biophys. Acta* 1819 137–148. 10.1016/j.bbagrm.2011.05.001 21605713 PMC3175014

[B81] Kim M. C. Chung W. S. Yun D. J. Cho M. J. (2009). Calcium and calmodulin-mediated regulation of gene expression in plants. *Mol. Plant* 2 13–21. 10.1093/mp/ssn091 19529824 PMC2639735

[B82] Kim T. H. Böhmer M. Hu H. Nishimura N. Schroeder J. I. (2010). Guard cell signal transduction network: advances in understanding abscisic acid, CO_2_, and Ca2+ signaling. *Annu. Rev. Plant Biol.* 61 561–591. 10.1146/annurev-arplant-042809-112226 20192751 PMC3056615

[B83] Kleinhenz M. D. Palta J. P. (2002). Root zone calcium modulates the response of potato plants to heat stress. *Physiol. Plant.* 115 111–118. 10.1034/j.1399-3054.2002.1150113.x 12010474

[B84] Klis F. M. Boorsma A. De Groot P. W. J. (2006). Cell wall construction in *Saccharomyces cerevisiae*. *Yeast* 23 185–202. 10.1002/yea.1349 16498706

[B85] Koch K. E. (1996). Carbohydrate-modulated gene expression in plants. *Annu. Rev. Plant Physiol. Plant Mol. Biol.* 47 509–540. 10.1146/annurev.arplant.47.1.509 15012299

[B86] Kohorn B. D. Johansen S. Shishido A. Todorova T. Martinez R. Defeo E. (2009). Pectin activation of MAP kinase and gene expression is WAK2 dependent. *Plant J.* 60 974–982. 10.1111/j.1365-313X.2009.04016.x 19737363 PMC3575133

[B87] Kohorn B. D. Kobayashi M. Johansen S. Riese J. Huang L. F. Koch K. (2006). An *Arabidopsis* cell wall-associated kinase required for invertase activity and cell growth. *Plant J.* 46 307–316. 10.1111/j.1365-313X.2006.02695.x 16623892

[B88] Komarova T. V. Pozdyshev D. V. Petrunia I. V. Sheshukova E. V. Dorokhov Y. L. (2014). Pectin methylesterase-generated methanol may be involved in tobacco leaf growth. *Biochemisrt (Mosc.)* 79 102–110. 10.1134/S0006297914020035 24794725

[B89] Konno H. Yamasaki Yoshiki Sugimoto M. Takeda K. (2008). Differential changes in cell wall matrix polysaccharides and glycoside-hydrolyzing enzymes in developing wheat seedlings differing in drought tolerance. *J. Plant Physiol.* 165 745–754. 10.1016/j.jplph.2007.07.007 17765362

[B90] Kumar S. V. Wigge P. A. (2010). H2A.Z-containing nucleosomes mediate the thermosensory response in *Arabidopsis*. *Cell* 140 136–147. 10.1016/j.cell.2009.11.006 20079334

[B91] Kwon Y. Kim S. H. Jung M. S. Kim M. S. Oh J. E. Ju H. W. (2006). *Arabidopsis* hot2 encodes an endochitinase-like protein that is essential for tolerance to heat, salt and drought stresses. *Plant J.* 49 184–193. 10.1111/j.1365-313X.2006.02950.x 17156413

[B92] Lämke J. Bäurle I. (2017). Epigenetic and chromatin-based mechanisms in environmental stress adaptation and stress memory in plants. *Genome Biol.* 18:124. 10.1186/s13059-017-1263-6 28655328 PMC5488299

[B93] Larkindale J. Hall J. D. Knight M. R. Vierling E. (2005). Heat stress phenotypes of *Arabidopsis* mutants implicate multiple signaling pathways in the acquisition of thermotolerance. *Plant Physiol.* 138 882–897. 10.1104/pp.105.062257 15923322 PMC1150405

[B94] Larkindale J. Vierling E. (2008). Core genome responses involved in acclimation to high temperature. *Plant Physiol.* 146 748–761. 10.1104/pp.107.112060 18055584 PMC2245833

[B95] Le Gall H. Philippe F. Domon J. M. Gillet F. Pelloux J. Rayon C. (2015). Cell wall metabolism in response to abiotic stress. *Plants (Basel)* 4 112–166. 10.3390/plants4010112 27135320 PMC4844334

[B96] Leroux C. Bouton S. Kiefer-Meyer M.-C. Fabrice T. N. Mareck A. Guénin S. (2015). PECTIN METHYLESTERASE48 is involved in Arabidopsis pollen grain germination. *Plant Physiol.* 167 367–380. 10.1104/pp.114.250928 25524442 PMC4326738

[B97] Levesque-Tremblay G. Müller K. Mansfield S. D. Haughn G. W. (2015). HIGHLY METHYL ESTERIFIED SEEDS is a pectin methyl esterase involved in embryo development. *Plant Physiol.* 167 725–737. 10.1104/pp.114.255604 25572606 PMC4348785

[B98] Li B. Liu H. T. Sun D. Y. Zhou R. G. (2004). Ca2+ and calmodulin modulate DNA-binding activity of maize heat shock transcription factor in vitro. *Plant Cell Physiol.* 45 627–634. 10.1093/pcp/pch074 15169945

[B99] Li H. Yan S. Zhao L. Tan J. Zhang Q. Gao F. (2014). Histone acetylation associated up-regulation of the cell wall related genes is involved in salt stress induced maize root swelling. *BMC Plant Biol.* 14:105. 10.1186/1471-2229-14-105 24758373 PMC4005470

[B100] Li H. Zhou S. Y. Zhao W. S. Su S. C. Peng Y. L. (2009). A novel wall-associated receptor-like protein kinase gene, OsWAK1, plays important roles in rice blast disease resistance. *Plant Mol. Biol.* 69:337. 10.1007/s11103-008-9430-5 19039666

[B101] Li X. Li Y. Qu M. Xiao H. Feng Y. Liu J. (2016). Cell wall pectin and its methyl-esterification in transition zone determine Al resistance in cultivars of pea (*Pisum sativum*). *Front. Plant Sci.* 7:39. 10.3389/fpls.2016.00039 26870060 PMC4734104

[B102] Li W. Shang H. Ge Q. Zou C. Cai J. Wang D. (2016). Genome-wide identification, phylogeny, and expression analysis of pectin methylesterases reveal their major role in cotton fiber development. *BMC Genomics* 17:1000. 10.1186/s12864-016-3365-z 27927181 PMC5142323

[B103] Lima R. B. Dos Santos T. B. Vieira L. G. Ferrarese Mde L. Ferrarese-Filho O. Donatti L. (2013). Heat stress causes alterations in the cell-wall polymers and anatomy of coffee leaves (*Coffea arabica* L.). *Carbohydr. Polym.* 93 135–143. 10.1016/j.carbpol.2012.05.015 23465912

[B104] Lin J. S. Kuo C. C. Yang I. C. Tsai W. A. Shen Y. H. Lin C. C. (2018). MicroRNA160 modulates plant development and heat shock protein gene expression to mediate heat tolerance in *Arabidopsis*. *Front. Plant Sci.* 9:68. 10.3389/fpls.2018.00068 29449855 PMC5799662

[B105] Lindquist S. Craig E. A. (1988). The heat-shock proteins. *Annu. Rev. Genet.* 22 631–677. 10.1146/annurev.ge.22.120188.003215 2853609

[B106] Liners F. Gaspar T. Van Cutsem P. (1994). Acetyl- and methyl-esterification of pectins of friable and compact sugar-beet calli: consequences for intercellular adhesion. *Planta* 192 545–556. 10.1007/BF00203593

[B107] Lionetti V. Cervone F. Bellincampi D. (2012). Methyl esterification of pectin plays a role during plant-pathogen interactions and affects plant resistance to diseases. *J. Plant Physiol.* 169 1623–1630. 10.1016/j.jplph.2012.05.006 22717136

[B108] Lionetti V. Raiola A. Camardella L. Giovane A. Obel N. Pauly M. (2007). Overexpression of pectin methylesterase inhibitors in *Arabidopsis* restricts fungal infection by *Botrytis cinerea*. *Plant Physiol.* 143 1871–1880. 10.1104/pp.106.090803 17277091 PMC1851811

[B109] Lionetti V. Raiola A. Cervone F. Bellincampi D. (2014). Transgenic expression of pectin methylesterase inhibitors limits tobamovirus spread in tobacco and *Arabidopsis*. *Mol. Plant Pathol.* 15 265–274. 10.1111/mpp.12090 24127644 PMC6638747

[B110] Liu H. C. Liao H. T. Charng Y. Y. (2011). The role of class A1 heat shock factors (HSFA1s) in response to heat and other stresses in *Arabidopsis*. *Plant Cell Environ.* 34 738–751. 10.1111/j.1365-3040.2011.02278.x 21241330

[B111] Liu H. T. Li B. Shang Z. L. Li X. Z. Mu R. L. Sun D. Y. (2003). Calmodulin is involved in heat shock signal transduction in wheat. *Plant Physiol.* 132 1186–1195. 10.1104/pp.102.018564 12857801 PMC167059

[B112] Liu H. T. Li G. L. Chang H. Sun D. Y. Zhou R. G. Li B. (2007). Calmodulin-binding protein phosphatase PP7 is involved in thermotolerance in *Arabidopsis*. *Plant Cell Environ.* 30 156–164. 10.1111/j.1365-3040.2006.01613.x 17238907

[B113] Liu H. T. Sun D. Y. Zhou R. G. (2005). Ca2+ and AtCaM3 are involved in the expression of heat shock protein gene in *Arabidopsis*. *Plant Cell Environ.* 28 1276–1284. 10.1111/j.1365-3040.2005.01365.x

[B114] Liu J. Feng L. Li J. He Z. (2015). Genetic and epigenetic control of plant heat responses. *Front. Plant Sci.* 6:267. 10.3389/fpls.2015.00267 25964789 PMC4408840

[B115] Lootens D. Capel F. Durand D. Nicolai T. Boulenguer P. Langendorff V. (2003). Influence of pH, Ca concentration, temperature and amidation on the gelation of low methoxyl pectin. *Food Hydrocoll.* 17 237–244. 10.1016/S0268-005X(02)00056-5

[B116] Louvet R. Cavel E. Gutierrez L. Guénin S. Roger D. Gillet F. (2006). Comprehensive expression profiling of the pectin methylesterase gene family during silique development in *Arabidopsis thaliana*. *Planta* 224 782–791. 10.1007/s00425-006-0261-9 16622707

[B117] Ma L. Xu X. Cui S. Sun D. (1999). The presence of a heterotrimeric G protein and its role in signal transduction of extracellular calmodulin in pollen germination and tube growth. *Plant Cell* 11 1351–1363. 10.1105/tpc.11.7.1351 10402434 PMC144279

[B118] Manabe Y. Nafisi M. Verhertbruggen Y. Orfila C. Gille S. Rautengarten C. (2011). Loss-of-function mutation of REDUCED WALL ACETYLATION2 in *Arabidopsis* leads to reduced cell wall acetylation and increased resistance to *Botrytis cinerea*. *Plant Physiol.* 155 1068–1078. 10.1104/pp.110.168989 21212300 PMC3046569

[B119] Matsunaga T. Ishii T. Matsumoto S. Higuchi M. Darvill A. Albersheim P. (2004). Occurrence of the primary cell wall polysaccharide rhamnogalacturonan II in pteridophytes, lycophytes, and bryophytes. Implications for the evolution of vascular plants. *Plant Physiol.* 134 339–351. 10.1104/pp.103.030072 14671014 PMC316313

[B120] Mellerowicz E. J. Gorshkova T. A. (2012). Tensional stress generation in gelatinous fibres: a review and possible mechanism based on cell-wall structure and composition. *J. Exp. Bot.* 63 551–565. 10.1093/jxb/err339 22090441

[B121] Micheli F. (2001). Pectin methylesterases: cell wall enzymes with important roles in plant physiology. *Trends Plant Sci.* 6 414–419. 10.1016/S1360-1385(01)02045-3 11544130

[B122] Min L. Li Y. Hu Q. Zhu L. Gao W. Wu Y. (2014). Sugar and auxin signaling pathways respond to high-temperature stress during anther development as revealed by transcript profiling analysis in cotton. *Plant Physiol.* 164 1293–1308. 10.1104/pp.113.232314 24481135 PMC3938621

[B123] Mittler R. Finka A. Goloubinoff P. (2012). How do plants feel the heat? *Trends Biochem. Sci.* 37 118–125. 10.1016/j.tibs.2011.11.007 22236506

[B124] Mlynárová L. Nap J. P. Bisseling T. (2007). The SWI/SNF chromatin-remodeling gene AtCHR12 mediates temporary growth arrest in *Arabidopsis thaliana* upon perceiving environmental stress. *Plant J.* 51 874–885. 10.1111/j.1365-313X.2007.03185.x 17605754

[B125] Mohnen D. (2008). Pectin structure and biosynthesis. *Curr. Opin. Plant Biol.* 11 266–277. 10.1016/j.pbi.2008.03.006 18486536

[B126] Mollet J. -C. Leroux C. Dardelle F. Lehner A. (2013). Cell wall composition, biosynthesis and remodeling during pollen tube growth. *Plants* 2 107–147. 10.3390/plants2010107 27137369 PMC4844286

[B127] Monroy A. F. Dhindsa R. S. (1995). Low-temperature signal transduction: induction of cold acclimation-specific genes of alfalfa by calcium at 25 degrees C. *Plant Cell* 7 321–331. 10.2307/3869854 7734966 PMC160785

[B128] Moore J. P. Nguema-Ona E. Chevalier L. Lindsey G. G. Brandt W. F. Lerouge P. (2006). Response of the leaf cell wall to desiccation in the resurrection plant *Myrothamnus flabellifolius*. *Plant Physiol.* 141 651–662. 10.1104/pp.106.077701 16603665 PMC1475438

[B129] Morimoto R. I. (2008). Proteotoxic stress and inducible chaperone networks in neurodegenerative disease and aging. *Genes Dev.* 22 1427–1438. 10.1101/gad.1657108 18519635 PMC2732416

[B130] Moscatiello R. Mariani P. Sanders D. Maathuis F. J. M. (2006). Transcriptional analysis of calcium-dependent and calcium-independent signalling pathways induced by oligogalacturonides. *J. Exp. Bot.* 57 2847–2865. 10.1093/jxb/erl043 16868046

[B131] Mosser D. D. Kotzbauer P. T. Sarge K. D. Morimoto R. I. (1990). In vitro activation of heat shock transcription factor DNA-binding by calcium and biochemical conditions that affect protein conformation. *Proc. Natl. Acad. Sci. U.S.A.* 87 3748–3752. 10.1073/pnas.87.10.3748 2339118 PMC53980

[B132] Nakamura A. Furuta H. Maeda H. Takao T. Nagamatsu Y. (2002). Structural studies by stepwise enzymatic degradation of the main backbone of soybean soluble polysaccharides consisting of galacturonan and rhamnogalacturonan. *Biosci. Biotechnol. Biochem.* 66 1301–1313. 10.1271/bbb.66.1301 12162553

[B133] Nonomura A. M. Benson A. A. (1992). The path of carbon in photosynthesis: improved crop yields with methanol. *Proc. Natl. Acad. Sci. U.S.A.* 89 9794–9798. 10.1073/pnas.89.20.9794 1409701 PMC50219

[B134] Ochoa-Villarreal M. Aispuro-Hernández E. Vargas-Arispuro I. Martínez-Téllez M. Á. (2012). “Plant cell wall polymers: function, structure and biological activity of their derivatives,” in *Polymerization* Chap. 4 ed. Gomes A. D. S. (Rijeka: InTech).

[B135] Ohama N. Sato H. Shinozaki K. Yamaguchi-Shinozaki K. (2017). Transcriptional regulatory network of plant heat stress response. *Trends. Plant Sci.* 22 53–65. 10.1016/j.tplants.2016.08.015 27666516

[B136] Osakabe Y. Osakabe K. Shinozaki K. Tran L. S. (2014). Response of plants to water stress. *Front. Plant Sci.* 5:86. 10.3389/fpls.2014.00086 24659993 PMC3952189

[B137] Osorio S. Castillejo C. Quesada M. A. Medina-Escobar N. Brownsey G. J. Suau R. (2008). Partial demethylation of oligogalacturonides by pectin methyl esterase 1 is required for eliciting defence responses in wild strawberry (*Fragaria vesca*). *Plant J.* 54 43–55. 10.1111/j.1365-313X.2007.03398.x 18088306

[B138] Peaucelle A. Braybrook S. A. Le Guillou L. Bron E. Kuhlemeier C. Höfte H. (2011). Pectin-induced changes in cell wall mechanics underlie organ initiation in *Arabidopsis*. *Curr. Biol.* 21 1720–1726. 10.1016/j.cub.2011.08.057 21982593

[B139] Pelletier S. Van Orden J. Wolf S. Vissenberg K. Delacourt J. Ndong Y. A. (2010). A role for pectin de-methylesterification in a developmentally regulated growth acceleration in dark-grown *Arabidopsis* hypocotyls. *New Phytol.* 188 726–739. 10.1111/j.1469-8137.2010.03409.x 20819179

[B140] Pelloux J. Rustérucci C. Mellerowicz E. J. (2007). New insights into pectin methylesterase structure and function. *Trends Plant Sci.* 12 267–277. 10.1016/j.tplants.2007.04.001 17499007

[B141] Philippe F. Pelloux P. Rayon C. (2017). Plant pectin acetylesterase structure and function: new insights from bioinformatic analysis. *BMC Genomics* 18:456. 10.1186/s12864-017-3833-0 28595570 PMC5465549

[B142] Pilling J. Willmitzer L. Fisahn J. (2000). Expression of a petunia inflata pectin methyl esterase in *Solanum tuberosum* L. enhances stem elongation and modifies cation distribution. *Planta* 210 391–399. 10.1007/PL00008147 10750896

[B143] Qu T. Liu R. Wang W. An L. Chen T. Liu G. (2011). Brassinosteroids regulate pectin methylesterase activity and *AtPME41* expression in Arabidopsis under chilling stress. *Cryobiology* 63 111–117. 10.1016/j.cryobiol.2011.07.003 21819976

[B144] Raiola A. Lionetti V. Elmaghraby I. Immerzeel P. Mellerowicz E. J. Salvi G. (2011). Pectin methylesterase is induced in *Arabidopsis* upon infection and is necessary for a successful colonization by necrotrophic pathogens. *Mol. Plant Microbe. Interact.* 24 432–440. 10.1094/MPMI-07-10-0157 21171891

[B145] Rana R. M. Dong S. N. Tang H. J. Ahmad F. Zhang H. S. (2012). Functional analysis of OsHSBP1 and OsHSBP2 revealed their involvement in the heat shock response in rice (*Oryza sativa* L.). *J. Exp. Bot.* 63 6003–6016. 10.1093/jxb/ers245 22996677

[B146] Renew S. Heyno E. Schopfer P. Liszkay A. (2005). Sensitive detection and localization of hydroxyl radical production in cucumber roots and *Arabidopsis* seedlings by spin trapping electron paramagnetic resonance spectroscopy. *Plant J.* 44 342–347. 10.1111/j.1365-313X.2005.02528.x 16212611

[B147] Rhee S. Y. Osborne E. Poindexter P. D. Somerville C. R. (2003). Microspore separation in the quartet 3 mutants of *Arabidopsis* is impaired by a defect in a developmentally regulated polygalacturonase required for pollen mother cell wall degradation. *Plant Physiol.* 133 1170–1180. 10.1104/pp.103.028266 14551328 PMC281612

[B148] Ridley B. L. O’Neill M. A. Mohnen D. (2001). Pectins: structure, biosynthesis, and oligogalacturonide-related signaling. *Phytochemistry* 57 929–967. 10.1016/S0031-9422(01)00113-3 11423142

[B149] Rombouts F. M. Thibault J. F. (1986). Enzymatic and chemical degradation and the fine structure of pectins from sugar-beet pulp. *Carbohydr. Res.* 154 189–203. 10.1016/S0008-6215(00)90032-6

[B150] Rose J. K. C. Braam J. Fry S. C. Nishitani K. (2002). The XTH family of enzymes involved in xyloglucan endotransglucosylation and endohydrolysis: current perspectives and a new unifying nomenclature. *Plant Cell Physiol.* 43 1421–1435. 10.1093/pcp/pcf171 12514239

[B151] Rui Y. Xiao C. Yi H. Kandemir B. Wang J. Z. Puri V. M. (2017). POLYGALACTURONASE INVOLVED IN EXPANSION3 functions in seedling development, rosette growth, and stomatal dynamics in *Arabidopsis thaliana*. *Plant Cell* 29 2413–2432. 10.1105/tpc.17.00568 28974550 PMC5774581

[B152] Ryden P. Sugimoto-Shirasu K. Smith A. C. Findlay K. Reiter W. D. Mccann M. C. (2003). Tensile properties of *Arabidopsis* cell walls depend on both a xyloglucan cross-linked microfibrillar network and rhamnogalacturonan II-borate complexes. *Plant Physiol.* 132 1033–1040. 10.1104/pp.103.021873 12805631 PMC167041

[B153] Saidi Y. Finka A. Muriset M. Bromberg Z. Weiss Y. G. Maathuis F. J. M. (2009). The heat shock response in moss plants is regulated by specific calcium-permeable channels in the plasma membrane. *Plant Cell* 21 2829–2843. 10.1105/tpc.108.065318 19773386 PMC2768932

[B154] Sato H. Mizoi J. Tanaka H. Maruyama K. Qin F. Osakabe Y. (2014). *Arabidopsis* DPB3-1, a DREB2A interactor, specifically enhances heat stress-induced gene expression by forming a heat stress-specific transcriptional complex with NF-Y subunits. *Plant Cell* 26 4954–4973. 10.1105/tpc.114.132928 25490919 PMC4311209

[B155] Satyal S. H. Chen D. Y. Fox S. G. Kramer J. M. Morimoto R. I. (1998). Negative regulation of the heat shock transcriptional response by HSBP1. *Genes Dev.* 12 1962–1974. 10.1101/gad.12.13.1962 9649501 PMC316975

[B156] Scharf K. D. Rose S. Zott W. Schöffl F. Nover L. (1990). Three tomato genes code for heat stress transcription factors with a region of remarkable homology to the DNA-binding domain of the yeast HSF. *EMBO J.* 9 4495–4501. 10.1002/j.1460-2075.1990.tb07900.x 2148291 PMC552242

[B157] Sénéchal F. L’enfant M. Domon J. M. Rosiau E. Crépeau M. J. Surcouf O. (2015). Tuning of pectin methylesterification: pectin methylesterase inhibitor 7 modulates the processive activity of co-expressed pectin methylesterase 3 in a pH-dependent manner. *J. Biol. Chem.* 290 23320–23335. 10.1074/jbc.M115.639534 26183897 PMC4645611

[B158] Sénéchal F. Wattier C. Rustérucci C. Pelloux J. (2014). Homogalacturonan-modifying enzymes: structure, expression, and roles in plants. *J. Exp. Bot.* 65 5125–5160. 10.1093/jxb/eru272 25056773 PMC4400535

[B159] Sentenac H. Grignon C. (1981). A model for predicting ionic equilibrium concentrations in cell walls. *Plant Physiol.* 68 415–419. 10.1104/pp.68.2.415 16661927 PMC427501

[B160] Shin S. B. Golovkin M. Reddy A. S. N. (2014). A pollen-specific calmodulin-binding protein, NPG1, interacts with putative pectate lyases. *Sci. Rep.* 4:5263. 10.1038/srep05263 24919580 PMC4053719

[B161] Siedlecka A. Wiklund S. Peronne M. A. Micheli F. Lesniewska J. Sethson I. (2008). Pectin methyl esterase inhibits intrusive and symplastic cell growth in developing wood cells of *Populus*. *Plant Physiol.* 146 554–565. 10.1104/pp.107.111963 18065553 PMC2245829

[B162] Solecka D. Żebrowski J. Kacperska A. (2008). Are pectins involved in cold acclimation and de-acclimation of winter oil-seed rape plants? *Ann. Bot.* 101 521–530. 10.1093/aob/mcm329 18222909 PMC2710196

[B163] Somssich M. Khan G. A. Persson S. (2016). Cell wall heterogeneity in root development of *Arabidopsis*. *Front. Plant Sci.* 7:1242. 10.3389/fpls.2016.01242 27582757 PMC4987334

[B164] Stief A. Altmann S. Hoffmann K. Pant B. D. Scheible W. R. Bäurle I. (2014). *Arabidopsis* miR156 regulates tolerance to recurring environmental stress through SPL transcription factors. *Plant Cell* 26 1792–1807. 10.1105/tpc.114.123851 24769482 PMC4036586

[B165] Sun X. T. Li B. Zhou G. M. Tang W. Q. Bai J. Sun D. Y. (2000). Binding of the maize cytosolic hsp70 to calmodulin, and identification of calmodulin-binding site in hsp70. *Plant Cell Physiol.* 41 804–810. 10.1093/pcp/41.6.804 10945351

[B166] Taketa S. Yuo T. Tonooka T. Tsumuraya Y. Inagaki Y. Haruyama N. (2012). Functional characterization of barley betaglucanless mutants demonstrates a unique role for CslF6 in (1,3;1,4)-β-D-glucan biosynthesis. *J. Exp. Bot.* 63 381–392. 10.1093/jxb/err285 21940720 PMC3245474

[B167] Talmadge K. W. Keegstra K. Bauer W. D. Albersheim P. (1973). The structure of plant cell walls: I. The macromolecular components of the walls of suspension-cultured sycamore cells with a detailed analysis of the pectic polysaccharides. *Plant Physiol.* 51 158–173. 10.1104/pp.51.1.158 16658279 PMC367375

[B168] Tenhaken R. (2014). Cell wall remodeling under abiotic stress. *Front. Plant Sci.* 5:771. 10.3389/fpls.2014.00771 25709610 PMC4285730

[B169] Tian G. W. Chen M. H. Zaltsman A. Citovsky V. (2006). Pollen-specific pectin methylesterase involved in pollen tube growth. *Dev. Biol.* 294 83–91. 10.1016/j.ydbio.2006.02.026 16564517

[B170] Tucker M. R. Lou H. Aubert M. K. Wilkinson L. G. Little A. Houston K. (2018). Exploring the role of cell wall-related genes and polysaccharides during plant development. *Plants* 7:42. 10.3390/plants7020042 29857498 PMC6028917

[B171] Turbant A. Fournet F. Lequart M. Zabijak L. Pageau K. Bouton S. (2016). PME58 plays a role in pectin distribution during seed coat mucilage extrusion through homogalacturonan modification. *J. Exp. Bot.* 67 2177–2190. 10.1093/jxb/erw025 26895630 PMC4809284

[B172] Uddin M. N. Hanstein S. Leubner R. Schubert S. (2013). Leaf cell-wall components as influenced in the first phase of salt stress in three maize (*Zea mays* L.) hybrids differing in salt resistance. *J. Agron. Crop Sci.* 199 405–415. 10.1111/jac.12031

[B173] Vierling E. (1991). The roles of heat shock proteins in plants. *Annu. Rev. Plant Physiol. Plant Mol. Biol.* 42 579–620. 10.1146/annurev.pp.42.060191.003051

[B174] Virdi A. S. Thakur A. Dutt S. Kumar S. Singh P. (2009). A sorghum 85 kDa heat stress-modulated protein shows calmodulin-binding properties and cross-reactivity to anti-*Neurospora crassa* Hsp 80 antibodies. *FEBS Lett.* 583 767–770. 19174162 10.1016/j.febslet.2009.01.025

[B175] Vogel J. (2008). Unique aspects of the grass cell wall. *Curr. Opin. Plant Biol.* 11 301–307. 10.1016/j.febslet.2009.01.025 18434239

[B176] Volpi C. Janni M. Lionetti V. Bellincampi D. Favaron F. D’ovidio R. (2011). The ectopic expression of a pectin methyl esterase inhibitor increases pectin methyl esterification and limits fungal diseases in wheat. *Mol. Plant Microbe Interact.* 24 1012–1019. 10.1016/j.pbi.2008.03.002 21585271

[B177] Von Dahl C. C. Hävecker M. Schlögl R. Baldwin I. T. (2006). Caterpillar-elicited methanol emission: a new signal in plant-herbivore interactions? *Plant J.* 46 948–960. 10.1094/MPMI-01-11-0021 16805729

[B178] Wagner T. A. Kohorn B. D. (2001). Wall-associated kinases are expressed throughout plant development and are required for cell expansion. *Plant Cell* 13 303–318. 10.1111/j.1365-313X.2006.02760.x 11226187 PMC102244

[B179] Wang L. C. Wu J. R. Chang W. L. Yeh C. H. Ke Y. T. Lu C. A. (2013). *Arabidopsis* HIT4 encodes a novel chromocentre-localized protein involved in the heat reactivation of transcriptionally silent loci and is essential for heat tolerance in plants. *J. Exp. Bot.* 64 1689–1701. 10.1105/tpc.13.2.303 23408827

[B180] Wang T. McFarlane H. E. Persson S. (2016). The impact of abiotic factors on cellulose synthesis. *J. Exp. Bot.* 67 543–552. 10.1093/jxb/ert030 26552883

[B181] Wen F. Zhu Y. Hawes M. C. (1999). Effect of pectin methylesterase gene expression on pea root development. *Plant Cell* 11 1129–1140. 10.1105/tpc.11.6.1129 10368183 PMC144245

[B182] Willats W. G. Orfila C. Limberg G. Buchholt H. C. Van Alebeek G. J. Voragen A. G. (2001). Modulation of the degree and pattern of methyl-esterification of pectic homogalacturonan in plant cell walls. Implications for pectin methyl esterase action, matrix properties, and cell adhesion. *J. Biol. Chem.* 276 19404–19413. 10.1074/jbc.M011242200 11278866

[B183] Wolf S. Mouille G. Pelloux J. (2009). Homogalacturonan methyl-esterification and plant development. *Mol. Plant* 2 851–860. 10.1093/mp/ssp066 19825662

[B184] Wu H. C. Hsu S. F. Luo D. L. Chen S. J. Huang W. D. Lur H. S. (2010). Recovery of heat shock-triggered released apoplastic Ca2+ accompanied by pectin methylesterase activity is required for thermotolerance in soybean seedlings. *J. Exp. Bot.* 61 2843–2852. 10.1093/jxb/erq121 20444907 PMC2882276

[B185] Wu H. C. Huang Y. C. Stracovsky L. Jinn T. L. (2017). Pectin methylesterase is required for guard cell function in response to heat. *Plant Signal. Behav.* 12:e1338227. 10.1080/15592324.2017.1338227 28617153 PMC5566256

[B186] Wu H. C. Jinn T. L. (2010). Heat shock-triggered Ca2+ mobilization accompanied by pectin methylesterase activity and cytosolic Ca2+ oscillation are crucial for plant thermotolerance. *Plant Signal. Behav.* 5 1252–1256. 10.4161/psb.5.10.12607 20948293 PMC3115360

[B187] Wu H. C. Jinn T. L. (2012). Oscillation regulation of Ca2+/calmodulin and heat-stress related genes in response to heat stress in rice (*Oryza sativa* L.). *Plant Signal. Behav.* 7 1056–1057. 10.4161/psb.21124 22899079 PMC3489625

[B188] Wu H. C. Luo D. L. Vignols F. Jinn T. L. (2012). Heat shock-induced biphasic Ca2+ signature and OsCaM1-1 nuclear localization mediate downstream signalling in acquisition of thermotolerance in rice (*Oryza sativa* L.). *Plant Cell Environ.* 35 1543–1557. 10.1111/j.1365-3040.2012.02508.x 22428987

[B189] Xiao C. Anderson C. (2013). Roles of pectin in biomass yield and processing for biofuels. *Front. Plant Sci.* 4:67. 10.3389/fpls.2013.00067 23543255 PMC3608898

[B190] Xiong J. Yang Y. Fu G. Tao L. (2015). Novel roles of hydrogen peroxide (H2O2) in regulating pectin synthesis and demethylesterification in the cell wall of rice (*Oryza sativa*) root tips. *New Phytol.* 206 118–126. 10.1111/nph.13285 25615266

[B191] Xu J. Tian J. Belanger F. C. Huang B. (2007). Identification and characterization of an expansin gene AsEXP1 associated with heat tolerance in C3 *Agrostis grass* species. *J. Exp. Bot.* 58 3789–3796. 10.1093/jxb/erm229 17928368

[B192] Yang C. M. Heilman J. L. (1991). Short-term high temperature effects on stomatal behaviors of rice plants. ?. Occurring at the grain-filling stage. *J. Agric. Res. China* 40 243–247.

[B193] Yang K. A. Lim C. J. Hong J. K. Park C. Y. Cheong Y. H. Chung W. S. (2006). Identification of cell wall genes modified by a permissive high temperature in Chinese cabbage. *Plant Sci.* 171 175–182. 10.1016/j.plantsci.2006.03.013

[B194] Yang S. Huang C. Wu Z. Hu J. Li T. Liu S. (2006). Stomatal movement in response to long distance-communicated signals initiated by heat shock in partial roots of *Commelina communis* L. *Sci. China Life Sci.* 49 18–25. 10.1007/s11427-005-0117-8 16544572

[B195] Yang T. Poovaiah B. W. (2002). A calmodulin-binding/CGCG box DNA-binding protein family involved in multiple signaling pathways in plants. *J. Biol. Chem.* 277 45049–45058. 10.1074/jbc.M207941200 12218065

[B196] York W. S. Kumar Kolli V. S. Orlando R. Albersheim P. Darvill A. G. (1996). The structures of arabinoxyloglucans produced by solanaceous plants. *Carbohydr. Res.* 285 99–128. 10.1016/S0008-6215(96)90176-7 9011379

[B197] Yu E. Fan C. Yang Q. Li X. Wan B. Dong Y. (2014). Identification of heat responsive genes in *Brassica napus* siliques at the seed-filling stage through transcriptional profiling. *PLoS One* 9:e101914. 10.1371/journal.pone.0101914 25013950 PMC4094393

[B198] Zhu J. Lee B. H. Dellinger M. Cui X. Zhang C. Wu S. (2010). A cellulose synthase-like protein is required for osmotic stress tolerance in *Arabidopsis*. *Plant J.* 63 128–140. 10.1111/j.1365-313X.2010.04227.x 20409003 PMC3061338

